# Prognostic and Clinicopathological Significance of SERTAD1 in Various Types of Cancer Risk: A Systematic Review and Retrospective Analysis

**DOI:** 10.3390/cancers11030337

**Published:** 2019-03-08

**Authors:** Raj Kumar Mongre, Samil Jung, Chandra Bhushan Mishra, Beom Suk Lee, Shikha Kumari, Myeong-Sok Lee

**Affiliations:** 1 Molecular Cancer Biology Laboratory, Cellular Heterogeneity Research Center, Department of Biosystem, Sookmyung Women’s University, Hyochangwon gil-52, Yongsan-Gu, Seoul 140-742, Korea; rajkmgr1208@gmail.com (R.K.M.); jj-31@hanmail.net (S.J.); min9996@nate.com (B.S.L.); 2 Dr. B. R. Ambedkar Center for Biomedical Research, University of Delhi, Delhi 110007, India; chandra.medicinalchemist@gmail.com (C.B.M.); shikha.pharma08@gmail.com (S.K.)

**Keywords:** SERTAD1, overall survival, disease/relapse free survival, mutation, correlation, protein interaction, meta-analysis, miRNAs

## Abstract

SERTAD/TRIP-Br genes are considered as a key nuclear transcriptional player in diverse mechanisms of cell including carcinogenesis. The Oncomine^™^-Online Platform was used for differential expression and biological insights. Kaplan-Meier survival estimated by KM-plotter/cBioPortal/PrognoScan with 95% CI. SERTAD1 was found significantly elevated levels in most of tumor samples. Kaplan-Meier Plotter results distinctly showed the SERTAD1 over-expression significantly reduced median overall-survival (OS) of patients in liver (*n* = 364/Logrank-test *p* = 0.0015), ovarian (*n* = 655/Logrank-test *p* = 0.00011) and gastric (*n* = 631/Logrank-test *p* = 0.1866). Increased level of SERTAD1 has a significantly higher survival rate in the initial time period, but after 100 months slightly reduced OS (*n* = 26/Logrank-test *p* = 0.34) and RFS in HER2 positive breast cancer patients. In meta-analysis, cancer patients with higher SERTAD1 mRNA fold resulted worse overall survival than those with lower SERTAD1 levels. Heterogeneity was observed in the fixed effect model analysis DFS [Tau^2^ = 0.0.073, *Q* (df = 4) = 15.536 (*p* = 0.004), I^2^ = 74.253], DSS [Tau^2^ = 1.015, *Q* (df = 2) = 33.214, (*p* = 0.000), I^2^ = 93.973], RFS [Tau^2^ = 0.492, *Q* (df = 7) = 71.133 (*p* = 0.000), I^2^ = 90.159] (Figure 5). OS [Tau^2^ = 0.480, *Q* (df = 17) = 222.344 (*p* = 0.000), I^2^ = 92.354]. Lastly, SERTAD1 involved in several signaling cascades through interaction and correlation with many candidate factors as well as miRNAs. This meta-analysis demonstrates a robust evidence of an association between higher or lower SERTAD1, alteration and without alteration of SERTAD1 in cancers in terms of survival and cancer invasiveness.

## 1. Introduction

The SERTAD or TRIP-Br nuclear factor family (known as TRIP-Br/p34SEI/SEI) play a vital role in neuronal cell proliferation, viral infections, and cancer progression [1,2,3,4]. The SERTAD family has recently been shown to modulate E2F-dependent transcriptional activities [5,6,7]. This family members include TRIPBr1/p34SEI-1/SERTAD1/SEI-1, TRIP-Br2/SERTAD2/SEI-2, TRIP-Br3/ HEPP/CDCA4/SEI-3, RBT1/SERTAD3 (henceforth referred to as RBT1) and the recently-identified as SERTAD4 [1,3,7,8]. Among them, SERTAD1 is considered as a key participant in numerous molecular processes. Recently, SERTAD1 have been demonstrated to be localized in tandem within a 19q13 amplicon frequently found in diverse human tumors, consistent with their putative role as oncogenes that promote tumor development [9]. Indeed, cytogenetic studies have exposed a gain of chromosomal region 19q13.1-13.2 in more than 30% of ovarian carcinomas as well as a numerous type of tumors including pancreatic carcinomas as well as lung cancers [10]. For instance, our earlier study revealed SERTAD1 is an oncoprotein that significantly contributes in oncogenesis and programmed cell death (PCD) under the nutrient and starvation condition [5]. It influenced cell growth through interaction with E2F1/DP-1 and cell regulator CDK4/p16INK4a [6,7,8]. An enhanced level of SERTAD1 remarkably induced tumor induction, ubiquitination and genomic instabilities [11,12,13]. It has been proved that an appropriate expression of SERTAD1 is essential to maintain cells and homeostasis. On the other hand, lower level of SERTAD1 displayed a binding interaction between HIC domain of I-mfa and SERTA domain [14]. In addition, I-mfa also took part in the transcription process of Fbxw7, which influenced by E2F/DP-1 and SERTAD1 [14]. I-mfa and SERTA domain containing proteins modulate several signaling cascades and may contribute to initiate benign tumor. Similarly, as described above, I-mfa and HIC both associated with C-terminal of SERTAD1 and affect trans-activating functions of SERTAD1 [14]. Adenylys cyclases (ACs) are essential enzymes that produce cyclic AMP, a regulator of cellular phenomenon [15]. One of the prominent finding showed SERTAD1 acts as an adaptor in the interaction of multiple AC with X-linked inhibitor of apoptosis protein (XIAP) and RING-domain E3 ubiquitin ligase [16]. Our previous finding also illustrated that N-terminus of SERTAD1 interacts with BIR2 domain of XIAP [17]. Additionally, SERTAD1 promotes cell survival through ubiquitination of apoptosis signal regulating kinase1 (ASK1) in response to ROS induction, ASK1 is involved in various cellular processes [12]. Collectively, the functional analysis of SERTAD1 showed putative functions with various mechanisms as reported in literature. However, distinct link between SERTAD1, tumor initiation and its pathogenesis is still remained to explain extensively. In the current meta-analysis, we wish to decipher the potential role of SERTAD1 as a tumorigenic factor as well as transcriptomic and translational biomarker for various types of human cancers.

The p16^INK4a^ acts as a growth factor sensor in the cell survival program and pathways [18]. A study has shown that SERTAD1 antagonizes cell cycle regulators through activation of p16^INK4a^ in quiescent fibroblasts [19]. It negatively regulates E2F transcription factor by regulating p16^INKAa^ during G1/S phase of cell cycle [1]. The inhibition of SERTAD1 results cell proliferation arrest in human nasopharyngeal cancer (CNE2), cervical cancer (CaSki) and melanoma (MeWo) cancer cell lines [3]. These findings clearly established SERTAD1 as a cell cycle modulator. Apart from that, SERTAD1 protein divided into various domains with distinct functions. Ablation of the PHD-binding domain and the C-terminal acidic rich domain of SERTAD1 regulates p53-intrinsic transcriptional activities [1,20]. In extension, PHD-bromo domain of Kruppel-associated box (KRAB) is associated with SERTAD1 and suppresses KRIP-1 (TIF1 alpha and beta)/p300/CBP functions. Some potential genes influence role of SERTAD1 in several biological phenomenon. According to previous reports, the retinoblastoma gene (RB) and adenovirus E1A oncogene modulate the action of E2F-1/DP-1 and SERTAD1/KRIP-1 [1]. Additionally, retinoblastoma protein pathway (pRb1-cyclinD1-cdk4/6-p16INK4A) also involved in SERTAD1 mediated regulation [21]. Moreover, the B-alpha isoform of PP2A (serine/threonine protein phosphatase 2A) controls cell proliferation through an association between PP2A-AB-alphaC holoenzyme and SERTAD1 [4]. Another report implied that SERTAD1 involves in Met induced formation of Double minute chromosomes (DMs). The DMs are associated with the chromosomal small fragment and abnormal cellular function in a high grade of tumor [22]. One more interesting finding declared that SERTAD1 promotes DMs formation by induction of PI3K/Akt/BRCA1-Abraxas pathway in NIH-3T3 fibroblasts [23]. 

Endogenous expression of SERTAD1 was observed in cells as well as in malignant tissues [24]. The normal expression of SERTAD1 can be modulated by many factors such as physical stress or starved environmental niche. Post-translational modifications in protein level remarkably promote abnormal SERTAD1 expression, which lead to augment programmed cell death [5,25]. Aberrant expression and persistent activation of SERTAD1 is implicated in neuronal cell proliferation, drug resistance against hypoxia-induced cell death and PCD [23,26]. It was found that over-expression of SERTAD1 is associated with the head and neck cancer [19]. Further, SERTAD1 differential expression also associated with numerous cancer types such as breast cancer, colon cancer, lung cancer, brain tumors, renal cancer, leukemia and lymphoma [24]. Even microarray based genes enrichment studies indicated the potential role of SERTAD1 but could not be confirmed by *in-vivo* studies till date. In the present study, we have generated genes networking and found putative targets to conduct xenograft paradigm for further elucidation. SERTAD1 targeted therapies might be proved effective and a novel approach to control various types of cancers. Therefore, we have also elicited the cancer patient’s survival analysis based on the higher or lower expression of SERTAD1 which may appear baseline for development of fabulous and novel treatment strategies for various types of cancers including breast cancer. However, the link between prognostic values of SERTAD1 in human tumors is still controversial. In this regard, we also carried out a systematic analysis combining thousands of genes expression or copy number variation analysis published online, to appraise the expression pattern, potential functions and distinct prognostic value of SERTAD1 in cancers.

## 2. Materials and Methods

### 2.1. Data Sources and Search Strategy, Selection, Data Extraction and Quality Assessment

Literature searches strategies were followed as reported previously [26]. Full text articles and datasets retrieved from Oncomine, cBioPortal online database, PubMed, Web of Science and Prognoscan online database system from 1999 to January 2019. Appropriate keywords to search were “SERTAD/TRIP-Br/SEI/p34SEI family in cellular function, relation between SERTAD1 gene expression and cancer progression (Oncomine/cBioPortal), mRNA expression of SERTAD1 associated with cancer patients survival (KM plotter/prognoscan), patients survival and hazard rate (HR) by cBioPortal/PrognoScan.” All selected literature were scrutinized according to previous report Newcastle-Ottawa Quality assessment Scale developed [27].

Selection of published articles and datasets for this meta-analysis based on “potential study that revealed significant association of SERTAD1/TRIP-Br1/SEI-1 in different types of cancer patients; reports investigated the association between SERTAD1 levels and survival outcomes in patients. In this regard, we also applied exclusion criteria: (a) reviews; (b) case reports or case series; (c) literature lacking enough information; (d) studies that do not match the final conclusion.

### 2.2. Transcriptomic and Differential Genes Expression Analysis

The Oncomine^™^ Platform—from web applications to translational bioinformatics services—provides solutions for individual researchers and multinational companies, with robust, peer-reviewed analysis methods and a powerful set of analysis functions that compute gene expression signatures, clusters and gene-set modules, automatically extracting biological insights from the data [24]. Online genomic profiling and analysis of SERTAD1 have been performed using Oncomine data base (https://www.oncomine.org/resource/login.html), that contains earlier reported data of microarray or RNA/DNA seq database. By using online web tool we performed identification and analysis of SERTAD1 which has been implicated in numerous cancers. Transcriptomic levels of SERTAD1 in tumor tissue were compared with that in normal control group using Student’s t-test to generate p-value (followed default settings of Oncomine). In addition, in Oncamine parameter was fixed as *p*-value < 0.0001, fold change >2, and gene ranking in the top 10% to get significantly mRNA levels of SERTAD1 probe. To confirm, the correlation between SERTAD1 and other genes have been defined by Heat map analysis in different types of cancers as previously reported [24].

### 2.3. Patients Survival Prediction: Retrospective Analysis

Survival of cancer patients can be affected by specific gene expression and alteration. Kaplan-Meier survival curve is one of the promising analytical method to predict survival and prognosis value. Kaplan-Meier Plotter (KM plotter) is an online database which contained microarray data sets [28]. Previous literatures revealed that KM-plotter (http://kmplot.com/analysis/) is an efficient to provide the role of 54,675 genes on survival using 10,461 cancer samples (5143 breast, 1816 ovarian, 2437 lungs and 1065 gastric cancer). In addition, the prognosis value of SERTAD1 has been assessed in patients bearing liver, ovarian, gastric, lung and breast cancer. 95% of confidence intervals (CI) and log rank *p*-value were opted to calculate significantly. 

### 2.4. Genetic Alteration Study, Patient Prognosis and Clinical Outcome: Meta-Analysis

Tumor biomarkers and therapeutic targets can be comprehensively accessed by PrognoScan (http://www.prognoscan.org/) online platform. “PrognoScan” is a tool for assessing the biological relationship between gene expression and prognosis. It contains a large collection of publicly available cancer microarray datasets with clinical annotation [29]. It opts the minimum *p*-value approach for grouping patients for overall survival (OS) and disease free survival (DFS) analysis that finds the optimal cutpoint in continuous gene expression measurement without prior fundamental concept, enables systematic meta-analysis of multiple cancer tissue microarray datasets. The clinical annotation using PrognoScan provides the opportunity for linking gene expression to prognosis.

Interactive exploration of multidimensional cancer genomics data sets have been mined and analyzed by cBioPortal, according to previous studies [30]. The cBioPortal is an open access resource (http://www.cbioportal.org/) that can analyze cancer genomics data for more than 5000 samples from 105 studies in TCGA pipeline [31]. To investigate various aspects of SERTAD1, we search in cBioPortal database and procured data. Gene manipulation, including amplification, deep deletion, missense mutations, copy number variance (CNV) data has been extracted with the default settings. OS and DFS were also measured based on online instruction of cBioPortal.

### 2.5. Tissue and Cancer Specific Biological Networks (TCSBN)

The database to explore the neighbors of a query gene have been analyzed as reported earlier [32]. The positive and negative correlation were elucidated using TCSBN followed default settings (Maximum number of nodes (min-1 max-200): 25, Edge Pruning Parameter (−log10 P) (min-0 max-50): 2). In addition, we generated tissue specific co-expression networks and integrated networks (INs) for breast, melanoma and liver cancers and compared with respect to its normal tissues. It is a novel database tool to explore the relationships between genes and biological functions in a given tissue/cancer through the employment of co-expression networks (CNs) in gene-centric manner.

### 2.6. Protein-Protein Interaction, Gene Common Pathways and miRNAs Association with SERTAD1

Protein–protein interaction has been confirmed and analyzed using the STRING bioinformatics online tool. STRING is a database of known and predicted protein-protein interactions [33]. Interactions in STRING are derived from five main sources as Genomic Context Predictions, High-throughput Lab Experiments, (Conserved) Co-Expression, Automated Textmining and Previous Knowledge in Databases. With the help of SERTAD1 query, we determined interaction between SERTAD1 and other interacted proteins. Associated protein network involved in various types of pathways has been investigated based on the default count in gene set and significant false discovery rate (FDR). Gene network construction and function prediction analyzed by GeneMANIA app according to previous literature [34].

Gene association and network derived using PCViz pathways commons network visualizer (http://www.pathwaycommons.org/pcviz/#neighborhood/SERTAD1). PCViz is efficient online platform to connect targets with interacted genes [35]. PCViz invented by Memorial Sloan-Kettering Cancer Center and University of Toronto. Datasets from PCViz extracted and analyzed by Cytoscape analyzing software tool as defined by somewhere [36]. Functional enrichment of potential microRNAs-SERTAD1 have been elucidated using Cytoscape.

### 2.7. Statistical Analysis

Cancer patient survival and prognostic values have been tested by the KM-plotter and Set significance threshold followed default settings as multiple hypothesis testing statistical method (*p* = 0.05), Bonferroni correction under 5% false discovery rate (FDR) [28,37]. Survival curves were plotted using the cBioPortal. All results are displayed with *p*-values from a long-rank test. Similarly, with Oncomine, heat maps. A *p*-values of <0.05 were considered to be statistically significant. In the Oncomine data analysis, *t*-Tests were conducted both as two-sided for differential expression analysis and one-sided for specific overexpression analysis. For the purpose of whole study analysis, *p*-values were corrected for multiple comparisons by the method of false discovery rates. Corrected *p*-values are designated as *Q* values [24], where *Q* = *P* × *n*/*i* (*n* = total number of genes; I = sorted rank of *p*-value).

In the meta-analysis, the pooled hazard ratios (HR) and 95% confidence intervals (CI) for the relationship between the number of patients with SERTAD1 mRNA expressing with overall, RFS, DSS and DFS survival were estimated using the DerSimonian and Laird (DL) random-effects and Mantel-Haenszel Fixed effect model to account for heterogeneity of study populations and designs [38]. The heterogeneity between the included studies was tested using the I^2^ index and Cochran’s *Q* test, with significant heterogeneity assumed for I^2^ > 50% or a *Q*-test *p*-value < 0.05 [39].

## 3. Results

### 3.1. Literature Search and Study Selection

**Figure 1:**
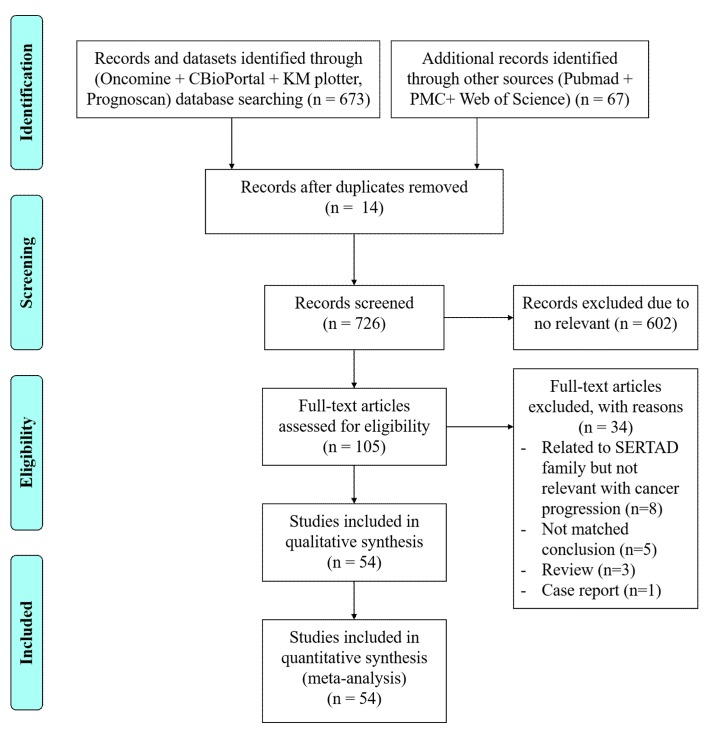
Flow chart of the selection process for the eligible studies for SERTAD1 retrospective study.

The strategies to search literature and study selection is summarized in Figure 1. A total 673 studies and datasets were selected from five online database system as Oncomine (*n* = 379 datasets and references), cBioPortal (*n* = 239), common pathway STRING (*n* = 4), KM plotter and TSBN (*n* = 2) and PrognoScan database (*n* = 34). Additional references and studies searched through PubMed, PMC and Web of Science (*n* = 67) selected for meta-analysis of SERTAD1. After reading of titles, abstracts and conclusions a total of 105 full text articles were retrieved for further evaluation. With further screening 54 articles included in qualitative and meta-analysis based on SERTAD1 expression and cancer patient clinical outcomes. For selection and search criteria of literature, we followed the guidelines of PRISMA.

### 3.2. Elevated Transcriptomic Levels of SERTAD1 Associated with Cancers

**Figure 2:**
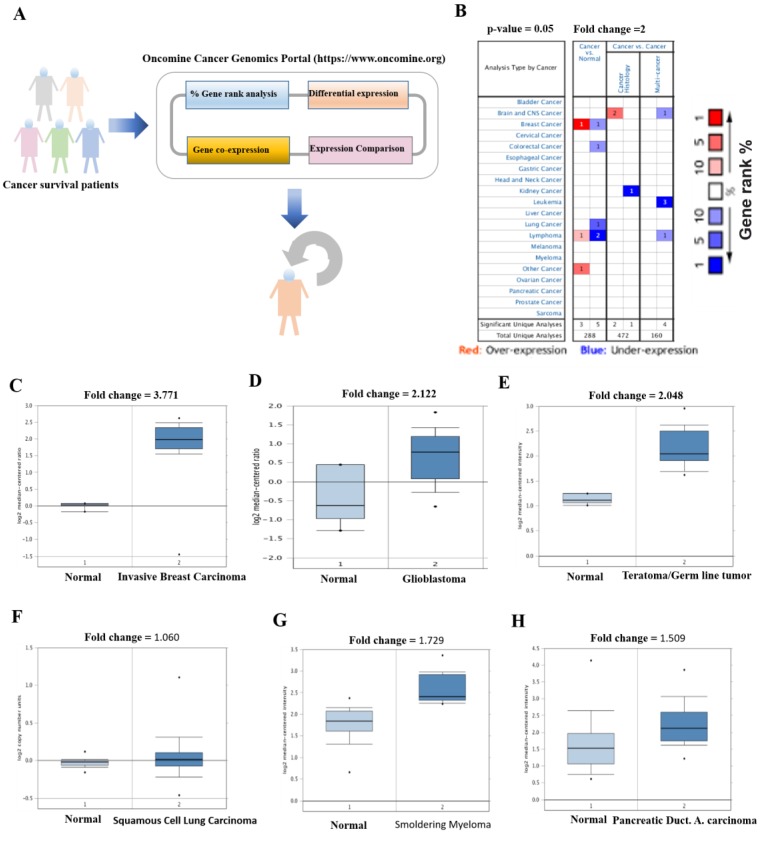
Elevated levels of SERTAD1 associated with cancers. (**A**) Schematic presentation of Oncomine analysis strategy from online genomics database, (**B**) Gene rank was calculated in tumor versus normal tissues. Table graphic was generated from Oncamine indicating the numbers of datasets with statistically (*p* < 0.01) mRNA over-expression (Red) or down-expression (Blue) of SERTAD1 (different types of cancers vs. corresponding normal tissue). The threshold was designed with following parameters *p*-value of *p* < 0.0001, fold change of 2, and gene ranking under 10% top genes. Table showed the fold change, *p*-value and rank of SERTAD1, (**C**) SERTAD1 Expression in Finak Breast cancer. Box-whisker plots of the gene expression of the most highly, moderate and low expressed SERTAD1 in Invasive Breast Carcinoma Stroma compared with corresponding normal breast tissues, (**D**) An elevated levels of SERTAD1 observed in germ line tumor with respect to its respective normal tissues. (**E**) mRNA levels of SERTAD1 in Astrocytoma and glioblastoma, (**F**–**H**) SERTAD1 mRNA fold changes in squamous lung, smoldering myeloma and pancreatic ductal adeno carcinoma with counterpart. Databased searched at *p* = 0.05, log2 median-centered, intensity, Gene rank based on 10% Top genes.

To evaluate the essential function of SERTAD1, we performed transcriptomic level analysis through the online database system Oncomine (https://www.oncomine.org, Figure 2A). Graphical table form results showed the Gene rank as a number of datasets with significant mRNA upregulated levels (Red) or suppressed (Blue) in cancer versus normal tissue (Figure 2B). Where, total number of unique analyses defining the threshold (*p* ≤ 0.05, fold change ≥ 2 and gene rank ≤ 10%) are depicted in the colored cells. This gene summary output gave us a clue to further elucidate the function of SERTAD1 in different types of cancers. Interestingly, we found that mRNA fold change of SERTAD1 was significantly elevated in tumor samples as compared to corresponding normal tissues as depicted in Figure 2C–H. As shown in Figure 2, SERTAD1 acts as a potential carcinogenic driver in most of the cancers such as invasive breast cancer, glioblastoma, teratoma, myeloma and pancreatic ductal carcinoma (Table 1). However, mRNA levels of SERTAD1 were not found significant as compared to normal tissue in squamous cell lung carcinoma (Figure 2F). In fact, differential expression revealed that lower and higher expression of SERTAD1 nuclear factor is associated with cancers like lung, breast blood and so on. Collectively, these data indicated a dire need to perform extensive survival analysis of cancer patients with respect to low and high expression of SERTAD1.

### 3.3. SERTAD1 Expression Define the Outcome of the Patient’s Survival in Cancers: A Meta-Analysis by KM-Plotter

The role of SERTAD1 in cancers has been widely studied as it has a dual role in carcinogenesis and patient survival. To retrospect function of SERTAD1 on the cancer patient’s outcome and survival, we investigated survival analysis based on its lower and higher expression considering default parameters of multiple hypothesis testing statistical method (*p* = 0.05), opted (according to KM-plotter guidelines) a Bonferroni correction threshold under 10% FDR to calculate significant analysis.

**Figure 3:**
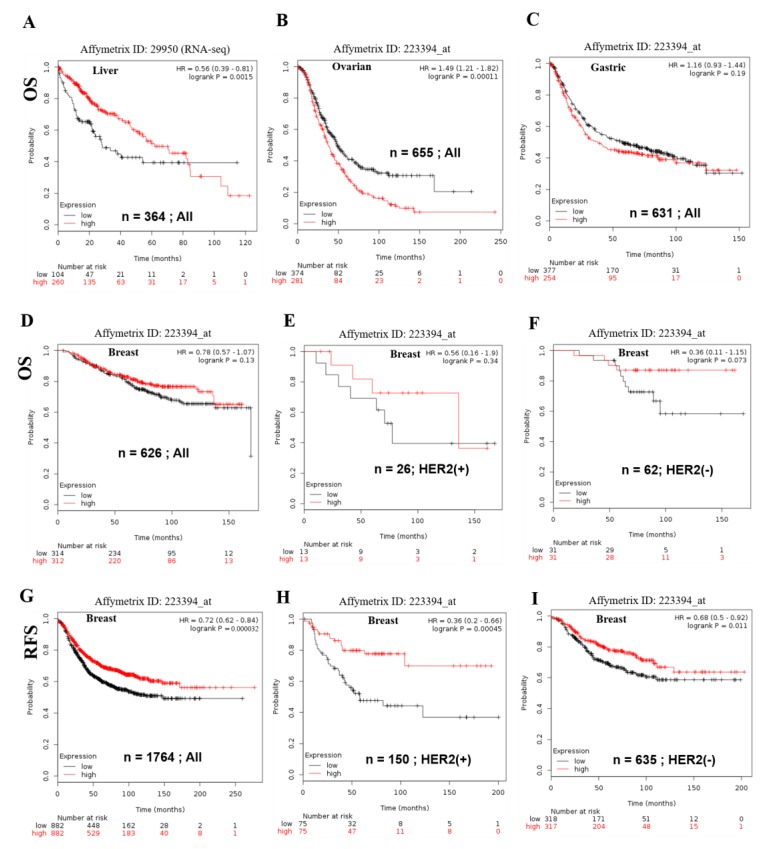
Kaplan-Meier overall survival curves for patients with different cancer cohort’s analysis. (**A**) Kaplan-Meier Survival plotter (KM-plotter) relationship between SERTAD1 expression and its effect on survival (*p* = 0.0015) on liver, (**B**) on ovarian (*p* = 0.00011), (**C**) on Gastric (*p* = 0.19), (**D**) breast (*p* = 0.13) cancer all, (**E**) HER2 (+) breast (*p* = 0.34) cancer, (**F**) HER2 (−) breast (*p* = 0.073) cancer, (**G**) Relapse free survival for breast (*p* = 0.000032) cancer all, (**H**) Relapse free survival for HER2 (+) breast (*p* = 0.00045) cancer, (**I**) Relapse free survival for HER2 (−) breast (*p* = 0.011) cancer. The *p*-values were calculated using the log-rank test. Vertical hash marks indicate censored data. The survival curve comparing the patient with high (red) and low (black) expression of SERTAD1.

Interestingly, Kaplan-Meier Plotter analysis distinctly showed the elevated expression significantly reduced patients median overall survival (OS) in liver (RNA seq data, *n* = 364, Logrank test *p* = 0.0015, HR = 0.56 and FDR 50%), ovarian (TCGA & GSE14764 data set, *n* = 655, Logrank test *p* = 0.00011, HR =1.49, FDR = 2%), gastric (GSE data set, *n* = 631, Logrank test *p* = 0.1866, HR = 1.16, FDR = 100%) and except in breast cancer (RNA seq data set, *n* = 626, Logrank test *p* = 0.1259, HR = 0.78) that shown in each Affymetrix ID (Figure 3A–D). On the other hand, the higher expression of SERTAD1 showed better outcome outcomes in relapse free survival (44.4 as compared to low exp. 31) of breast cancer patients (RNA seq data set, *n* = 626, Logrank test *p* = 0.000032, HR = 0.72). Human epidermal growth factor receptor 2 (HER2) plays the pivotal function in the breast cancer development and progression. To confirm key role of SERTAD1 in the presence of HER2, we elucidated Kaplan-Meier survival analysis as showed in Figure 3E–I. We found that patient groups with elevated levels of SERTAD1 gene (135.84 months) have a significantly higher survival rate as compared to lower expression holding patient groups (77.4) in the initial time period, but after 100 months a slightly reduced OS (*n* = 26, Logrank test *p* = 0.34, HR = 0.56) in HER2 positive patients is seen (Figure 3E). 

The findings demonstrated that overall survival was significantly higher in SERTAD1 over-expressing patients (*n* = 62, Logrank test *p* = 0.073, HR = 0.36) (Figure 3F). Treatment of breast cancer patients with different drugs in a time course manner also show better survival. The patients survive without any symptoms of cancer recurrence after treatment with anticancer drug that can be known as “relapse-free survival (RFS)”. Interestingly, SERTAD1 over-expressing group have shown higher RFS in HER2 positive patients as compared to HER2 negative (Figure 3G–I). PrognoScan database showed the prognostic value of SERTAD1 expression (Table 2). The poor prognosis has been seen in most of cancer patients, including brain, colon, lung, ovarian and skin cancer patients with higher SERTAD1 expression (Table 2 and Supplementary Figure S1) was in line with the obtained data from Kaplan-Meier plotter analysis (Figure 3). With the exception of different cancers, higher survival rate observed with higher levels of SERTAD1 in breast cancer patients. Apart from the expression of gene, mutation has also been proven as a key factor to augment in several types of cancers.

### 3.4. SERTAD1 Expression Associated with Patient’s Survival: Meta-Analysis by ProgonoScan Database

**Figure 4:**
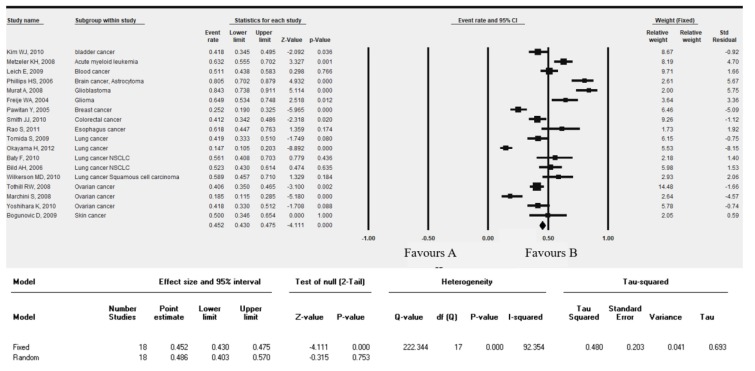
The association of SERTAD1 expression with patient’s survival and death hazard ratio (HR). Forest plot representing meta-analysis of SERTAD1 levels and its efficacious role in cancer invasiveness and clinical outcome. Effect sizes in the individual studies are indicated by the data markers, 95% confidence intervals are indicated by the error bars of HR.

To investigate the clinicopathological function of SERTAD1 mRNA levels in cancer patients, we collected studies for meta-analysis from PrognoScan database. SERTAD1 elevated mRNA levels significantly effects the overall patient survival in cancers (Figure 4 and Figure S1). Some of the meta-analysis (13 studies) showed a significant event rate in bladder (HR = 0.48; 95% CI = 0.345–0.495, *p* = 0.036), glioma (HR = 0.649; 95% CI = 0.534–0.748, *p* = 0.012), acute myeloid leukemia (HR = 0.632; 95% CI = 0.555–0.702, *p* = 0.001), colorectal (HR = 0.412; 95% CI = 0.342–0.486, *p* = 0.020) and ovarian (HR = 0.406; 95% CI = 0.350–0.465, *p* = 0.002) cancer. Moderate cancer risk was depicted in blood cancer (HR = 0.511; 95% CI = 0.438–0.583, *p* = 0.766) and NSCLC lung cancer (HR = 0.561; 95% CI = 0.408–0.703, *p* = 0.436). Lowest cancer risk was found in lung cancer (HR = 0.147; 95% CI = 0.105–0.203, *p* = 0.000) and ovarian cancer (HR = 0.185; 95% CI = 0.115–0.285, *p* = 0.000). Meta-analysis showed heterogeneity [Tau^2^ = 0.480, Q (df = 17) = 222.344 (*p* = 0.000), I^2^ = 92.354] in the fixed effect model (Figure 4).

**Figure 5:**
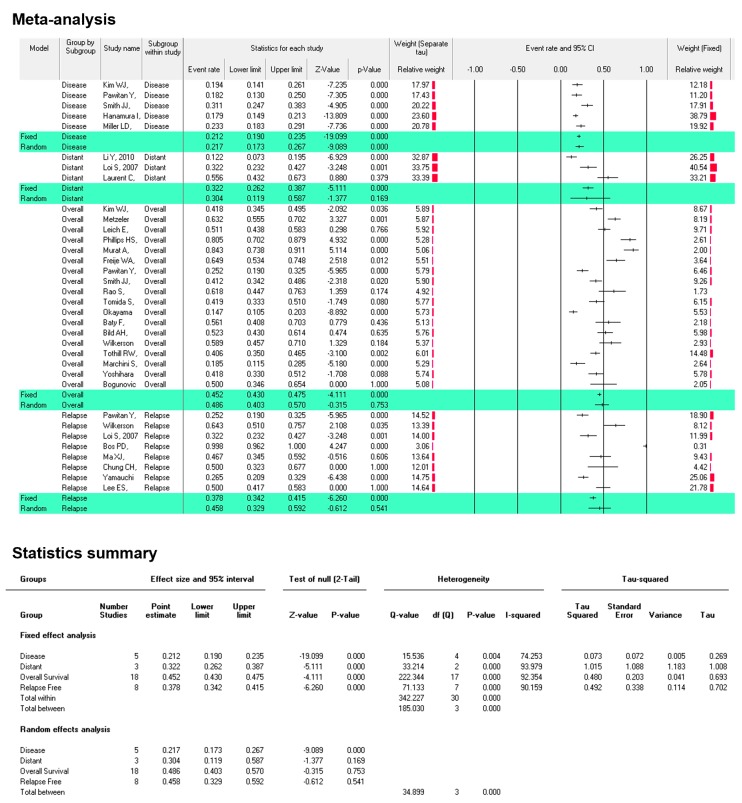
Event rate as cancer risk for overall, disease specific survival, relapse free survival, distant metastasis free survival based on Fixed and Random effect model. Meta-analysis: To further elucidate the comparison of hazard rate in order to OS, DSS, DFS and RFS. We have scrutinized selected 34 eligible studies according to SERTAD1 expression and patient’s survival.

We further elucidated cancer risk (hazard rate) based on SERTAD1 mRNA levels in term of OS, DFS, DSS and RFS. We selected 34 eligible studied from PrognoScan cancer micro-array datasets with clinical annotation. Results showed that cancer patients with higher SERTAD1 mRNA expression resulted worse overall survival in brain, colon, lung, blood and ovarian cancer than those with lower SERTAD1 levels (Figure 5 and Figure S1). Both random effect and fixed effect model analyzed in meta-analysis. Heterogeneity was observed in fixed effect model analysis that is DFS [Tau^2^ = 0.0.073, Q (df = 4) = 15.536 (*p* = 0.004), I^2^ = 74.253], DSS [Tau^2^ = 1.015, Q (df = 2) = 33.214, (*p* = 0.000), I^2^ = 93.973], RFS [Tau^2^ = 0.492, Q (df = 7) = 71.133 (*p* = 0.000), I^2^ = 90.159] (Figure 5).

### 3.5. Genetic Aberration in SERTAD1 Bestows More Invasive Cancers

Genetic mutation occurs due to several factors which always play a crucial role in the carcinogenesis. To identify the potential role of SERTAD1 after aberrant expression and mutation, we investigated the frequency of mutation using cBioPortal (http://www.cbioportal.org/index.do?session_id=5b611e29498eb8b3d567300e). Our result indicated that approximately 1.2% (768) cancer patient’s samples showed alteration of SERTAD1 in pathways and gene sets. 

**Figure 6:**
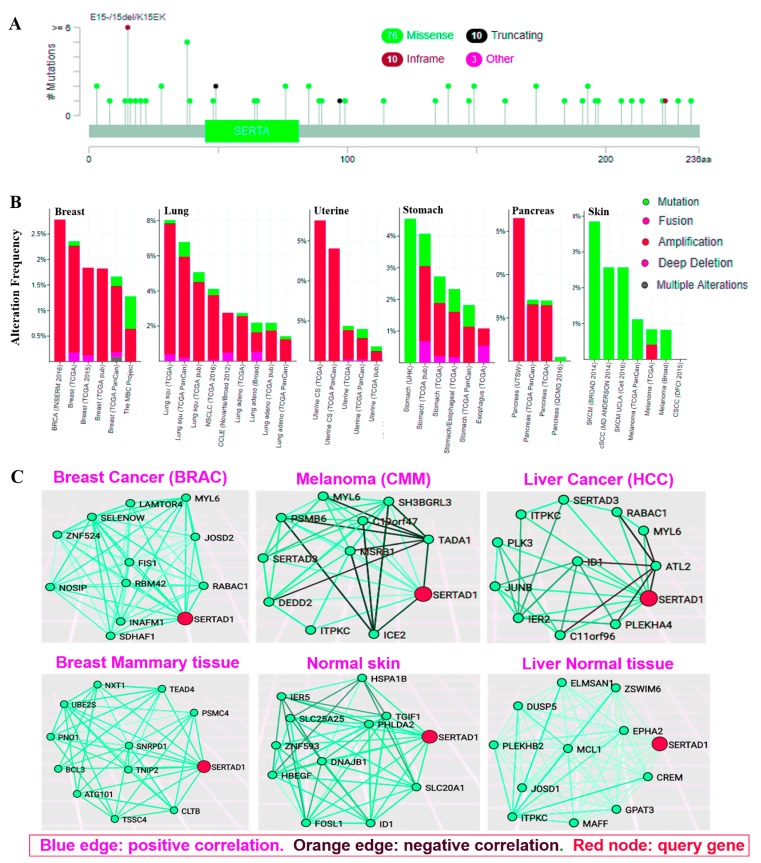
Aberrant transcribed and major mutation of SERTAD1 in different types of cancers across protein domains. (**A**) A total of 99 mutation sites were detected and located between amino acids 0 to 236 of SERTAD1. SERTAD-1 mutation mainly occurred in Pancreatic and uterine cancer. Moreover, hotspot area of mutation was found near SERTA and cycling binding domain, (**B**) The alteration frequency of a SERTAD1 gene was determined using cBIOPortal. Depicted cancer types containing >100 samples and alteration frequency of >15% are shown. The potential alteration frequency included deletions (Blue), amplification (Red), multiple alteration (Grey), or mutation (Green). The correlation between the alterations of SERTAD1, a putative target of cancer, across different cancer types. Data was obtained from the cBioportal for cancer genomics (Memorial Sloan-Kettering Cancer Center, New York, NY, USA). (**C**) Tissue and Cancer Specific Biological Network. SERTAD1 play a positive or negative regulator. Comparative network analysis showed crucial role of SERTAD1 breast cancer as positive regulator of all correlated genes in Normal and BRAC tumor (**C**. Left upper and bottom), Potential role of SERTAD1 in melanoma where it is inhibiting function of TADA1 and ICE2 (**C**. Middle upper and bottom). Co-expression networking of SERTAD1 showed as negative regulator for ALT2 in liver cancer HCC (**C**. Right upper and bottom). Gene MYL6 positively correlated by SERTAD1 in breast, melanoma and HCC. TCSBN network derived based on manual filtered maximum number of nodes and Edge Pruning Parameter (−log10 P) and (min-0 max-50): 2 respectively. The database to explore the neighbors of a query gene SERTAD1 (red color) have been analyzed.

A total of 103 mutation sites were detected, including missense, deletion, truncating and in-frame as well as amplification in different cancers (Figure 6A). The most mutation sites located between 0 and 236 amino acids while lung adenocarcinoma and stomach adenocarcinoma showed the mutation hotspot in cyclic A binding domain which is near to the SERTA domain of SERTAD1 (Figure 6A). In addition, specific target gene manipulation augments copy number alteration in most of the cancers. Therefore, we investigated SERTAD1 percentage alteration frequency with different types of cancers. In lung cancer, SERTAD1 analyzes in 3885 querying samples among eight studies that showed 163 queried samples which have been altered in the pathway and gene set [30,51,52].

Datasets exerted total gene alteration 28% (Mutation = 5.21%, amplification = 24.99%, Deep deletion = 1.24%). Datasets from 3989 samples (six studies) of breast cancer patients showed 76 (1.9%) queried samples with altered gene sets/pathway [31,53,54]. The total frequency of alteration of SERTAD1 around 11.71% (Mutation = 0.91%, amplification = 10.32%, Deep deletion = 0.39%). Uterine cancer datasets showed 1691 samples (five studies) and 72 (4.3%) of queried samples was found altered (Figure 6B and Table S1) [55]. In contrast, uterine cancer samples have total 39.93% mutation (Mutation = 1.63%, amplification = 37.88%, Deep deletion = 0.37%). Stomach cancer, results from 1235 samples (four studies) showed alteration in 34 (2.8%) of queried samples, frequency of alteration 13.16% (Mutation = 7.09%, amplification = 5.18%, Deep deletion = 0.89%) [56,57]. 935 samples (four studies) have been analyzed and found 46 (4.9%) of queried samples altered in Pancreas cancer that showed the frequency of alteration 31.01% (mutation = 1.52%, amplification = 29.48%, Deep deletion = 0.0%) [58,59]. Cohort studies of skin cancer also showed the alteration 9.81% (mutation = 9.39%, amplification = 0.42%, Deep deletion = 0.0%) [60] from 635 samples (four studies) in which nine (1.4%) samples was found altered (Figure 6B and Table S2). Abnormal expression of SERTAD1 links with several carcinogenic activities.

Following the current study by TCSBN correlation analysis of SERTAD1 in cancer and normal tissue, SERTAD1 significantly correlated with JOSD2 (Corr = 6.15) and UBE2S (Corr = 0.588) in breast tumor and normal tissue respectively (Figure 6C and Table S3). In skin tissue, SERTAD1 correlated with DEDD2 (Corr = 0.687) in melanoma and normal skin tissue. In addition, it is correlated with IER2 (Corr = 0.521) and PLEKHB2 (Corr = 0.832) in HCC and normal liver tissue respectively (Figure 6C and Tables S4 and S5). In contrast to the above findings, TCSBN depicted that SERTAD1 is negatively correlated with TADA1 (Corr = −0.624), ICE2 (Corr = −0.629) and ATL2 (Corr = −0.429) in melanoma and HCC respectively (Table S5). Collectively an altered or abnormal expression in the SERTAD1 correlated with different types of targets as compared to normal SERTAD1 expressing tissue. Therefore, aberration in SERTAD1 expression play a key role in the cancer patients’ survival.

### 3.6. The SERTAD1 Signature Prognosticate Better Outcome than Cases with Alteration: Meta-Analysis

**Figure 7:**
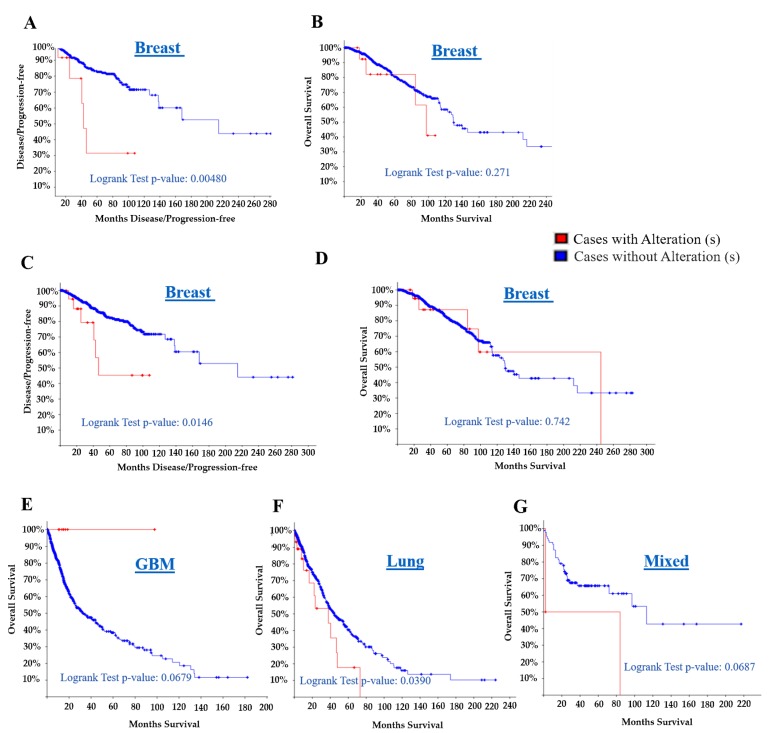
Kaplan-Meier Estimate for SERTAD1 alteration/mutation associated with poor prognosis and patient’s survival. (**A** & **B**) Breast Invasive Carcinoma (TCGA, Cell 2015) Tumor Samples with sequencing and CNA data (816 samples) (Gene Set/Pathway is altered in 15 (1.8%) of queried samples), (**C** & **D**) Breast Invasive Carcinoma (TCGA, Provisional) Tumor Samples with sequencing and CNA data (963 samples) (Gene Set/Pathway is altered in 23 (2.4%) of queried samples), (**E**) Merged Cohort of LGG and GBM (TCGA, Cell 2016) Tumor Samples with sequencing and CNA data (794 samples), Gene Set/Pathway is altered in 8 (1%) of queried samples, (**F**) Pan-Lung Cancer (TCGA, Nat Genet 2016) Tumor Samples with sequencing and CNA data (1144 samples), Gene Set/Pathway is altered in 47 (4.1%) of queried samples, (**G**) Mixed Tumors (PIP-Seq 2017) Sequenced Tumors (103 samples), altered in 3 (2.9%) of queried samples.

As explained earlier, expression as well as mutation directly affect tumour progression in cancer patients. In this line, we aimed to investigate whether the alteration in the SERTAD1 signature affects cancer relapse free and overall survival in different cohort studies. More interesting, breast cancer TCGA and CNA data revealed an alteration in 15 (1.8%) samples out of 816 samples. Figure 7A,B showed the patients with alteration of SERTAD1 have secured about 41.05% overall survival (Logrank test *p* = 0.271) compared to without altered patients group (65.93%). In disease free survival analysis, we observed significantly lower survival (42.81 months, Logrank test *p* = 0.0048) in SERTAD1 altered group with respect to cases without alterations (214.72 months) in breast cancer (Figure 7A and Table 3). One more study (TCGA, CNA data 23 samples have alteration out of 963) displayed similar findings as disease free survival significantly (*p* = 0.0146) lowest compared to without altered patients group (Figure 7A and Table 3). More interestingly, when the overall survival was tested on the subset (merged cohorts of LGG and GBM, TCGA datasets) of patients with altered SERTAD1 by sequence, we observed no significant reduction (*p* = 0.0679) which leads almost 100% patient survival in glioblastoma (Figure 7E). In addition, alteration of SERTAD1 is exhibiting greater likelihood of dying from glioblastoma as reciprocal to other cancers (Figure 7F and Table 3). Pan-Lung cancer TCGA and Mixed tumors PIP-seq datasets showed alteration of SERTAD1 significantly reduced overall survival of both lung (*p* = 0.0390) and mixed (*p* = 0.0687) cancer patients (Figure 7F,G). Here, we also confirmed the effect of the SERTAD1 alteration in cancer patient’s outcome and survival prognostic value (Table S6). Therefore, it is needed to find cellular mechanism and association of SERTAD1 with another other candidate factor to delineate essential functional pathways.

### 3.7. SERTAD1 Cross Talks with the Certain Candidate Targets: As Bridge Avenue Model

Differential expression of SERTAD1 simultaneously regulated its transcriptive change as well as tumor invasiveness, therefore we aimed to perform the meta-analysis that revealed expression of top 15% ranking SERTAD1 augmented genes which were studied in invasive breast carcinoma in 53 samples and compared with six normal breast tissue samples (Figure S2) as previously postulated in invasive breast cancer tissue across 53 publicly accessible breast cancer databases. Interestingly, SERTAD1 appeared as one of the top gene among the 19,189 genes which scored the rank 29 with a 3.77 fold change with significant *p*-value. With high *p*-value PARK2 also expressed similarly with 995 gene rank and 1.89 fold change. On the other hand, the expression pattern of small ubiquitin-like modifier 1 (SUMO1) and synuclein alpha (SNCA) have been also observed as vice versa between normal and invasive breast carcinoma tumor tissues. SUMO1 and SNCA both have −36.66 and −7.54 fold change with p-value 1.00 and their rank is much lower 18,612 and 18,972, respectively. These results suggested that negative relation between SERTAD1/PARK2 and SUMO1/SNCA which showed promising target signature to overcome from invasive breast carcinogenesis. One of the novel co-expression analysis of SERTAD1 also revealed significantly association with other genes in Oncomine datasets analysis [24], Illumina^®^ microarray system conducted and measured 17,409 genes in mesenchymal stem cells in response to Wnt3a treatment. Oncomine online datasets revealed various candidate genes, including nuclear factor interleukin 3 (NFIL3), ERBB Receptor Feedback Inhibitor 1 (ERRFI1) and Prostaglandin-Endoperoxide Synthase 2 (PTGS2) which showed higher correlation with 0.985, and 0.982 significant scores. This study represents a similar expression pattern of SERTAD1 in Wnt3a-CM incubation between 1 hour and 24 hours (Figure S2). In addition, another study of correlation showed SERTAD1 is significantly associated with Protein Phosphatase 1 Regulatory Subunit 15A (PPP1R15A), Early Growth Response 2 (EGR2), and NFKB Inhibitor Zeta (NFKBIZ) with a significantly correlation score 0.998, and 0.997 respectively. The Common pathway and Cytoscape approaches have determined that SERTAD1 is involving in various gene set pathways as TP53. Before investigate the correlation of SERTAD1 with other candidate genes between cancer and normal tissue, we detected SERTAD1 (highest 17%) mutated together with other genes through oncoprint analysis in uterine cancer (Figure S2). Independent studies also confirmed that SERTAD1 is correlated and interacted with many factors as well as miRNAs that involved in various cellular programs (Figure 6C, Figures S3 and S4 and Table S7) as parallel to earlier reports [16,17,64]. These tiny miRNAs and factors are able to form complex network to regulate cell fate, differentiation, development and homeostasis in normal and cancer cells.

## 4. Discussion

Earlier elucidation proved that the SERTAD family of nuclear transcriptional factors plays the pivotal function in most of mitogenic signaling pathways of cancers [1,3,65]. This family of proteins plays a crucial role in cell cycle management, gene transcriptional regulation as well as cancer pathogenesis [66]. Among SERTAD family SERTAD1 has a multiple function not only in neuronal cell death but also in carcinogenic processes [17,67]. SERTAD1 has been well studied as a transcriptional regulator, senescence inhibitor, cell cycle regulator, and apoptosis inhibitor [68]. Our results showed the elevated levels of SERTAD1 functioning as a cancer modulator, especially carcinogenic in nature in few cancers. These results similar to our earlier findings where SERTAD1 showed potential role in breast cancer development. An attempt to postulate the novel function of SERTAD1, we performed transcripts, changing analysis using “Oncomine Cancer Genome Portal (https://www.oncomine.org)” following previously reported study [24]. Oncomine dataset findings exhibited differential expression and gene rank of SERTAD1 (29 out of more than 1900 genes) among a number of sequenced genes in many cancers. This results lined with some novel studies that exhibited the potential role and gene rank of SERTAD1 in cancer progression [40,41,42,43,44,45,46,47,48,49,50]. Further, we dissected the protein expression of SERTAD1 and found SERTAD1 affects chemotherapeutic drug targeting human cancer via synthesized SERTAD1 decoy peptide in nasopharyngeal cancer (CNE2), Cervical cancer (CaSki) and melanoma (MeWo) cancer [3]. As our current findings, an elevated expression of SERTAD1 induces tumor development. Therefore, it is interesting to dissect the most essential function of SERTAD1 based on its low and high expression with regards to the patient’s survival and clinical outcome.

Candidate genes expression modulates cancer progression, metastasis and poor prognosis of patients [69]. The SERTAD1 signature associated with liver, ovarian, and gastric poor patient’s outcome. The Kaplan-Meier plotter is efficient to predict role of 54,675 genes on survival from 10,451 cancer samples and it is a better tool for meta-analysis for cancer biomarker [28,70]. Kaplan-Meier Plotter distinctly exhibited a higher expression of SERTAD1 significantly (Logrank test *p* = 0.0015, *n* = 364) reduced median overall survival (OS) of patients with liver cancer. And, lower expression of SERTAD1 predicted significant (*n* = 655, Logrank test *p* = 0.00011) enhanced overall survival of patients with ovarian cancer while there is no significant (*n* = 631, Logrank test *p* = 0.1866) differences observed in gastric cancer [29]. The most interesting fact is that we postulated expression analysis of SERTAD1 in breast cancer and we observed reciprocal results (high expression of SERTAD1 revealed higher overall survival, *n* = 626, Logrank test *p* = 0.1259) from other cancers. The findings evoked that HER2 positive exhibited higher overall/disease free survival with respect to HER2 negative sub-population. HER2 (human epidermal growth factor receptor 2) is a gene that plays a crucial role in the development of breast cancer and patient survival [71]. On the other hand, higher expression of SERTAD1 showed significantly better outcome in relapse free survival (44.4 as compared to low expression 31) of breast cancer patients (RNA seq data set, *n* = 626, Logrank test *p* = 0.000032, HR = 0.72). We also found that patient group with elevated levels of SERTAD1 gene (135.84 months) has the significantly higher survival rate with respect to lower expression group (77.4) in the initial time period but after 100 months slightly reduced OS (*n* = 26, Logrank test *p* = 0.34, HR = 0.56) in HER2 positive patients. SERTAD1 expressed in cancer tissue and control patient survival outcome. Our forest plot meta-analysis showed the cancer patient’s survival or hazard ratio is parallel to another report [70]. In addition, Forest plot meta-analysis showed over expression of SERTAD1 which responses, poor outcome of patients with varying hazard ratio at 95% CI. After investigation of expression pattern and its role in cancer induction, further, we had analyzed the effect of mutation of SERTAD1 on numerous types of cancers.

Expectedly we found that SERTAD1 is altered in most of the cancers, including lung (>8%), breast (>2.5), uterine (15%), stomach (4%), and pancreas (15%) as well as skin (3%) cancer (Figure 4B). Manipulation in gene resulted abnormal function is considered as a key factor in cancer development. Aberration in SERTAD1 studied using the cBioPortal online tool (http://www.cbioportal.org) as previously [30,31]. The cBio Cancer Genomics Portal is an open-access database system for interactive exploration of multidimensional cancer genomics and biologic insights as well as clinical applications. Total 768 (1.1%) of queried samples out of 67,857 samples have been shown an alteration in various pathways and gene sets. These finding suggested the mutation in SERTAD1 affects cancer induction by producing different types of alterations. Overall 103 mutational points have been observed in entire 236 amino acid sequence of SERTAD1. Among other domain, cyclicA binding domain showed mutation hotspot and several amino acids has been changed throughout the mutational process. Apart from that, lung cancer (163 out of 3885 samples) has been shown alteration frequency 28%, including Mutation, amplification and Deep deletion [30,51,52,63]. In case of breast cancer, total 76 samples showed alteration out of 3989 samples with 11.71% that also supported by past literature [31,53,54,61]. Similarly, uterine cancer datasets showed 39.93% mutation [55] and stomach cancer datasets showed 13.16% alteration frequency [56,57,72]. In the pancreas cancer, datasets showed a frequency of alteration 31.01% [58,59]. Least alteration frequency was observed in cohorts of skin cancer around 9.81% [60].

The effect of SERTAD1 alteration in cancer patient’s survival and their outcome has been also studied. Breast cancer TCGA and CNA sequenced data showed alteration around 1.8% and overall survival 41.05% (Logrank test *p* = 0.271) compared to without altered patient group (65.93%) and more disease free survival (Logrank test *p* = 0.00480) of patients. In fact, another breast cancer study also predicted similar disease free survival, significantly (*p* = 0.0146) reduced the alteration with respect to without altered patients complemented by previous findings [30,61]. On the other hand, in the subset (merged cohorts of LGG and GBM, TCGA datasets), we observed almost 100% patient survival of glioblastoma patients with altered SERTAD1. In addition, alteration of SERTAD1 exhibits greater possibility of dying from glioblastoma as reciprocal to other cancers [62]. Pan-Lung cancer PIP-seq datasets showed alteration of SERTAD1 significantly reduced both lung (*p* = 0.0390) and mixed (*p* = 0.0687) patients overall survival.

Differential expression of SERTAD1 is associated with a number of candidate genes in cancers as well as normal tissues. The correlation of SERTAD1 with genes of Drosophila as trithorax (trxG) and polycomb (PcG), contribute in an epigenetic modulation. If we look at structure of TARA protein of drosophila then we found that it is sharing conserved motifs with many mammalian nuclear proteins including SERTAD1 (TRIP-Br1), TRIP-Br2, Y127 and RBT1 [73]. These associated genes showed co-expression pattern in sequenced tumor samples. Especially SERTAD1 and Parkin2 have shown significantly higher fold change 3.77 and 1.89 in breast cancers tissue compared to normal tissue respectively [40]. On the other hand, transcriptomic level significantly opposed by SUMO1 and SNCA which are over-expressed in normal tissue. Further, after the expression analysis of SERTAD1 with other genes, we next tried to elucidate the putative function and correlation of SERTAD1 using tissue and cancer specific biological network (TCSBN). Nuclear transcription factor SERTAD1 is involved in many carcinogenesis processes through the interaction, association and correlation with the number of genes and performs as a key regulator in cancer progression. An attempt to elucidate the relation between nuclear factors SERTAD1 with other genes, we derived correlation of SERTAD1using Oncomine and TCSBN database as parallel to previous studies [24,32]. Interestingly, it predicted positive association in breast cancer through co-relation with MYL6, RABAC1 and other genes, while negatively regulating ATL2, ICE2 and TADA1 in melanoma and HCC. SERTAD1 associated and interacted with several proteins which have been confirmed by STRING protein analyzer. The STRING database is online protein-protein interaction and network predictor that potentially investigate proteins, physical as well as functional association [33]. There are multi-factors that regulate functions of SERTAD1 in different signaling cascades. Here, we found that cross talk of SERTAD1 not only with candidate proteins but also several microRNAs. As shown in Figure 6C, the SERTAD1 influenced by a number of microRNAs through acquired signals. MicroRNAs are key master regulators in many biological processes, including cancer progression [74]. These hypothetical analyses postulated novel paradigm through adopting “multi-proteins cross talk” or “gene/microRNAs bridge avenue model” to mitigate cancer development, recurrence and metastasis.

## 5. Concluding Remarks and Future Perspectives

SERTAD protein domains work as functional units and control gene networks as well as cell fate. Further, SERTAD1 protein consists four major types of domains such as cyclin-A binding domain, SERTA domain, PHD-bromo binding and C-terminal domains. The conserved cyclin-A binding domain defined functional interplay between anaphase-promoting complex CDKs and Cyclin-A-CDK4/6 cell cycle progression. Apart from that, it also involved in the regulation of cell cycle cyclin E, CDK4 activity, S-checkpoint and G2/M phase regulated in response to serum [7]. Although many findings revealed functions of SERTAD1, however, it is still unclear that how each protein domain regulates mechanism of cell proliferation in-vitro or ex-vivo. Distinct function of SERTAD1 to act as an oncogenic protein through specific signaling pathways yet is totally unclear whether and how this transcription factor promotes cancer metastasis expansion in an appropriate xenograft model. Answers to all such questions might come by the knock-in experiments in which the combined approach of “bridge between SERTAD1, oncogenes and microRNAs” will be adopted. Further, an essential paradigm to elaborate in-vivo phenotype analyzes can be effective molecular model to address the role of SERTAD1 protein activities by various types of post translational modification mechanisms. Moreover, now it seems that aberrant expression of SERTAD1 associated with some cancers including breast, liver, lungs and pancreatic, etc. Our analysis demonstrated that SERTAD1 was found significantly elevated in most of tumor samples. Kaplan-Meier Plotter results clearly showed the SERTAD1 over-expression significantly reduced median overall-survival (OS) of patients associated with liver, ovarian and gastric cancer. Enhanced level of SERTAD1 has a significantly higher survival rate in the initial time period, however, after 100 months slightly reduced OS and RFS in HER2 positive breast cancer patients. Additionally, our findings showed that there are several miRNAs regulating the transcriptional functions of SERTAD1. Despite of the significant progress in the investigation of the key role of SERTAD1 in XIAP or miRNAs mediated in cancer progression, few important issues remain to be experimentally addressed. Although astounding findings have been investigated in characterizing the potential role of the SERTAD1 with many oncoproteins targets as upstream modulator (CDKs), CCND1, XIAP and E2F1, but still mechanisms about how these pathway molecules modulate cancer cell program in patients model remain to be solved. However, distinct evidence of cross talk between SERTAD family (SERTAD1) and p53, STAT3, cancer pathways is difficult to obtain as it is necessarily obfuscated by deficits in the main cellular program in which a provided mechanism components functions. The same holds true for interpretation of the biological outcomes of deleting signaling molecules that may bridge SERTAD1 to other pathways and several miRNAs which are responsible for regulation of all biological processes. In this novel study, we have tried to correlate SERTAD1 and its transcriptional activities with many miRNAs as “Bridge Model” to make the innovative approach to ameliorate the inflammatory, metabolic, neuronal, bacterial/viral transmitted diseases and cancer. It is expected that application of SERTAD1 inhibitors in combination with miRNAs will produce more efficient killing of the tumorigenic cancer cells and may lead to slower or less recurrence of cancer. Another important aspect is that inhibition of SERTAD1 might target cancer not only directly by blocking anti-apoptosis mechanisms of malignant cells but also indirectly by shifting macrophages from the tumor-tolerating M2-polarization stage to the tumor attacking M1-stage by neoplasia suppressor miRNAs. Thus, these valuable findings and exhaustive discussion about role of SERTAD1 in cancer progression may open a new way for the management of numerous type of cancers. However, further research is needed to explore potential role, mechanisms, especially patient’s clinical outcome and prognosis of SERTAD1 in cancers as well as other diseases.

## Figures and Tables

**Table 1:** Differential expression of SERTAD1 in various types of cancer.

Cancer	Cancer Subtype	Fold Change	Rank %	Sample Size	Measured Genes	References
Breast cancer	Invasive breast carcinoma	3.771	29	59	19,189	[40]
Brain	Glioblastoma	2.122	199	54	14,836	[41]
Brain	Ganglioneuroma	3.576	245	64	19,574	[42]
Teratoma	Germ cell tumors	2.048	584	107	17,779	[43]
Blood	Lymphoma	2.192	1213	67	19,574	[44]
Blood	Leukemia	1.512	3409	2,096	19,574	[45]
Lung	Lung Adenocarcinoma	1.951	270	156	19,574	[46]
Blood	Smoldering Myeloma	1.729	1486	78	19,574	[47]
Lung	Squamous Cell Lung Carcinoma	1.060	5041	291	18,823	[48]
Pancreases	Pancreatic Ductal Adenocarcinoma	1.509	5174	78	19,574	[49]
Non-cancerous	Normal human tissues	3.200	1926	123	14,430	[50]

Oncomine online genomics database revealed the mRNA fold change of SERTAD1 in various cancer. Oncamine parameter was fixed as *p*-value < 0.0001, fold change > 2, and gene ranking in the top 10% to get significantly mRNA levels of SERTAD1 probe.

**Table 2:** The association of SERTAD1 transcriptomic levels with the survival in cancer patients.

Dataset	Cancer Type	Endpoint	Probe ID	*N*	COX *p*-Value	HR (95%CI)
GSE13507	Bladder cancer	Overall Survival	ILMN_1794017	165	0.251762	1.22
GSE13507	Bladder cancer	Disease Specific Survival	ILMN_1794017	165	0.242189	1.37
GSE12417-GPL97	Blood cancer	Overall Survival	223394_at	163	0.893883	1.03
GSE12417-GPL570	Blood cancer	Overall Survival	223394_at	79	0.668121	1.11
GSE16131-GPL97	Blood cancer	Overall Survival	223394_at	180	0.549863	1.21
GSE2658	Blood cancer	Disease Specific Survival	223394_at	559	0.185263	0.70
GSE4271-GPL97	Brain cancer	Overall Survival	223394_at	77	0.144382	1.39
GSE7696	Brain cancer	Overall Survival	223394_at	70	0.563036	0.84
GSE4412-GPL97	Brain cancer	Overall Survival	223394_at	74	0.149164	1.66
GSE16581	Brain cancer	Overall Survival	223394_at	67	0.223619	0.26
GSE19615	Breast cancer	Distant Metastasis Free Survival	223394_at	115	0.124646	0.22
GSE12276	Breast cancer	Relapse Free Survival	223394_at	204	0.171138	0.73
GSE6532-GPL570	Breast cancer	Relapse Free Survival	223394_at	87	0.494388	0.72
GSE6532-GPL570	Breast cancer	Distant Metastasis Free Survival	223394_at	87	0.494388	0.72
GSE9195	Breast cancer	Relapse Free Survival	223394_at	77	0.115978	0.33
GSE9195	Breast cancer	Distant Metastasis Free Survival	223394_at	77	0.029313	0.18
GSE1378	Breast cancer	Relapse Free Survival	7818	60	0.980828	1.01
GSE1379	Breast cancer	Relapse Free Survival	7818	60	0.400311	1.37
GSE1456-GPL97	Breast cancer	Disease Specific Survival	223394_at	159	0.864582	1.10
GSE1456-GPL97	Breast cancer	Overall Survival	223394_at	159	0.728309	0.84
GSE1456-GPL97	Breast cancer	Relapse Free Survival	223394_at	159	0.778333	1.15
GSE3494-GPL97	Breast cancer	Disease Specific Survival	223394_at	236	0.228813	1.91
GSE4922-GPL97	Breast cancer	Disease Free Survival	223394_at	249	0.276618	1.59
GSE17536	Colorectal cancer	Overall Survival	223394_at	177	0.861646	1.07
GSE17536	Colorectal cancer	Disease Specific Survival	223394_at	177	0.522633	1.31
GSE17536	Colorectal cancer	Disease Free Survival	223394_at	145	0.083306	2.36
GSE14333	Colorectal cancer	Disease Free Survival	223394_at	226	0.109716	1.51
GSE17537	Colorectal cancer	Overall Survival	223394_at	55	0.940023	1.04
GSE17537	Colorectal cancer	Disease Free Survival	223394_at	55	0.715296	0.80
GSE17537	Colorectal cancer	Disease Specific Survival	223394_at	49	0.781497	0.81
GSE11595	Esophagus cancer	Overall Survival	756322	34	0.960091	1.02
GSE22138	Eye cancer	Distant Metastasis Free Survival	223394_at	63	0.743321	1.08
GSE2837	Head and neck cancer	Relapse Free Survival	g12803668_3p_at	28	0.217278	1.60
GSE13213	Lung cancer	Overall Survival	A_23_P218463	117	0.598235	0.86
GSE31210	Lung cancer	Relapse Free Survival	223394_at	204	0.902867	1.05
GSE31210	Lung cancer	Overall Survival	223394_at	204	0.191555	1.89
GSE11117	Lung cancer	Overall Survival	H200004691	41	0.125025	1.49
GSE3141	Lung cancer	Overall Survival	223394_at	111	0.084274	1.48
GSE8894	Lung cancer	Relapse Free Survival	223394_at	138	0.214296	1.17
GSE17710	Lung cancer	Relapse Free Survival	25284	56	0.804400	1.05
GSE17710	Lung cancer	Relapse Free Survival	23819	56	0.892106	1.03
GSE17710	Lung cancer	Overall Survival	25284	56	0.742781	1.08
GSE17710	Lung cancer	Overall Survival	23819	56	0.797209	1.06
GSE9891	Ovarian cancer	Overall Survival	223394_at	278	0.097897	1.37
GSE8841	Ovarian cancer	Overall Survival	12603	81	0.258771	1.69
GSE17260	Ovarian cancer	Progression Free Survival	A_23_P218463	110	0.419954	1.14
GSE17260	Ovarian cancer	Overall Survival	A_23_P218463	110	0.384906	1.19
GSE19234	Skin cancer	Overall Survival	223394_at	38	0.429824	1.53

HR = Hazard ratio at 95% confidence interval, COX *P*-VALUE = Log rank test *p*-value, Prognostic survival results derived from PrognoScan online genomics datasets tool.

**Table 3:** Gene alteration of SERTAD1 regulates prognostic value of patient’s survival.

Study	Overall Survival Kaplan-Meier Estimate							Disease/Progression-Free Kaplan-Meier Estimate				
	Log Rank Test *p*-Value	Altheration/Mutation	Total No. of Cases	No. of Cases with Deceased	Median Months Survival	% of Survival	Survival Months	Log Rank Test *p*-Value	Altheration/Mutation	Total No. of Cases	No. of Cases with Relapsed	Median Months Disease-Free
A.	0.271	With	15	4	97.4	41.03	107.85	0.00480	With	13	5	42.81
		Without	799	114	129.6	65.94	234.10		Without	727	80	214.72
B.	0.742	With	23	5	244.91	59.78	244.91	0.0146	With	21	6	46.39
		Without	938	130	129.6	65.93	282.69		Without	858	96	214.72
C.	0.0679	With	8	0	NA	100	97.80					
		Without	721	263	32.4	24.64	182.20					
D.	0.382	With	2	2	35	50	109					
		Without	20	12	106	84.44	186					
E.	0.442	With	13	5	86.85	34.92	60.84	0.177	With	11	4	32.62
		Without	162	80	56.27	47.32	173.69		Without	110	39	61.6
F.	0.0687	With	2	2	2	50	84					
		Without	86	31	113	97.67	217					
G.	0.0390	With	40	13	37.83	17.77	73.16					
		Without	914	259	44.21	36.50	224.10					

Patient’s survival was obtained from cBioPortal multidimensional cancer genomics database. Each study revealed significant finding based on previous reported data sets. A Breast Invasive Carcinoma, TCGA, *Cell* 2015 [61], Tumor Samples with sequencing and CNA data (816 samples)/SERTAD1 Gene altered in 15 (1.8%) of queried samples; B Breast Invasive Carcinoma, TCGA, Provisional [30], Tumor Samples with sequencing and CNA data (963 samples)/SERTAD1 Gene altered in 23 (2.4%) of queried samples; C Merged Cohort of LGG and GBM, TCGA, *Cell* 2016 [62], Tumor Samples with sequencing and CNA data (794 samples)/SERTAD1 Gene altered in 8 (1%) of queried samples; D Low-Grade Gliomas, UCSF [30], Sequenced Tumors (61 samples)/SERTAD1 Gene altered in 2 (3.3%) of queried samples; E Lung Squamous Cell Carcinoma, TCGA, Provisional [30], Tumor Samples with sequencing and CNA data (178 samples)/SERTAD1 Gene altered in 13 (7.3%) of queried samples; F Mixed Tumors, PIP-Seq 2017 [30], Sequenced Tumors (103 samples)/SERTAD1 Gene altered in 3 (2.9%) of queried samples; G Pan-Lung Cancer, TCGA, *Nat. Genet.* 2016 [63], Tumor Samples with sequencing and CNA data (1144 samples)/SERTAD1 Gene altered in 47 (4.1%) of queried samples.

## Supplementary Materials

The following are available online at https://www.mdpi.com/2072-6694/11/3/337/s1, Figure S1: Relationship between SERTAD1 expression and patient outcome in different cancer patient’s survival analysis, Figure S2: Putative relationship and co-expression of SERTAD1 with various candidate factors, Figure S3: The bridge avenue model for SERTAD1 association and its relationship with genes, proteins and miRNAs, Figure S4: Transcription factor profiling of SERTAD1 promoter region, Table S1: Mutation of SERTAD1 in different types of cancers from cBioPortal.org, Table S2: Mutation in SERTAD1 in various types of cancers, Table S3: Tissue and cancer specific biological networks depicted significant correlation network between breast cancer and normal tissue, Table S4: Tissue and cancer specific biological networks depicted significant correlation network between Melanoma and normal skin tissue, Table S5: Tissue and cancer specific biological networks depicted significant correlation network between Liver cancer and normal liver tissue, Table S6: SERTAD1 expression and alteration versus cancer patient’s prognosis, Table S7: Associated protein network involved in various types of pathways.
