# Supplementary material for: Prognostic and Clinicopathological Significance of SERTAD1 in Various Types of Cancer Risk: A Systematic Review and Retrospective Analysis
Source: Cancers (Basel). 2019 Mar 8;11(3):337. doi: 10.3390/cancers11030337 (PMC6469047; doi:10.3390/cancers11030337)
Supplement: Supplementary file 1 [file cancers-11-00337-s001.pdf]

Supplementary Materials: Prognostic and Clinicopathological Significance of SERTAD1 in Various Types of Cancer Risk: A Systematic Review and Retrospective Analysis

Raj Kumar Mongre, Samil Jung, Chandra Bhushan Mishra, Beom Suk Lee, Shikha Kumari and Myeong-Sok Lee

Table S1. Mutation of SERTAD1 in different types of cancers from cBioPortal.org.

Cancer	Study [1]	Gene Altered %	Total Cases	Mutation %	Amplification %	Deep Deletion %
Lung	L. squ,TCGA	8.02	511	0.2	7.44	0.39
	L. squ, TCGA PanCan	6.78	487	0.82	5.75	0.21
	L. squ, TCGA Pub	5.06	178	0.56	4.49	-
	NSCLC, TCGA, 2016	4.11	1144	0.35	3.61	0.09
	L. adeno, TCGA	2.73	586	0.17	2.56	-
	L. adeno, TCGA, Broad	2.19	183	0.55	1.09	0.55
Breast	B. TCGA	2.35	1105	0.09	2.08	0.18
	B. TCGA, 2015	1.83	818	-	1.71	0.12
	B. TCGA, Pub	1.82	825	-	1.82	-
	B.TCGA, PanCan	1.66	1084	0.18	1.29	0.09
	B. MBC project	1.27	157	0.64	0.64	-
	BRCA, INSERM 2016	2.78	216	-	2.78	-
Uterine	U. CS, TCGA	17.54	57	-	17.54	-
	U. CS, TCGA, PanCan	14.04	57	-	14.04	-
	U. TCGA	4.38	548	0.55	3.65	0.18
	U. TCGA PanCan	3.97	529	1.13	2.65	0.19
Stomach	Stomach, UHK	4.55	22	4.55	-	-
	Stomach TCGA Pub	4.07	295	1.02	2.37	0.68
	Stomach TCGA	2.72	478	0.84	1.67	0.21
	Stomach TCGA PanCan	1.82	440	0.68	1.14	-
Pancreas	P. UTSW, Nat.com 2015	16.51	109	-	16.51	-
	P.TCGA, PanCan,	7.07	184	0.54	6.52	-
	P. TCGA provisional	6.99	186	0.54	6.45	-
	P. QCMG Nature 2016	0.44	456	0.44	-	-
Skin	SKCM, BROAD, 2014	3.85	78	3.85	-	-
	cSSC, MD ANDERSON 2014	2.56	39	2.56	-	-
	SKCM, UCLA, Cell 2016	2.56	39	2.56	-	-
	Melanoma TCGA	0.84	479	0.42	0.42	-

[1] All the studies are retrieved and analyzed using cBioPortal online database platform and followed as previous literature [30].

Table S2. Mutation in SERTAD1 in various types of cancers.

Study (Data retrieved from http://www.cbioportal.org/) [1]	Sample ID	Protein Change	Mutation Type	MS
Head and Neck Squamous Cell Carcinoma (TCGA)	TCGA-CV-7250-01	P38S	Missense_Mutation	Unknown
Skin Cutaneous Melanoma (Broad)	MEL-13567	P38S	Missense_Mutation	NA
Head and Neck Squamous Cell Carcinoma (TCGA, Provisional)	TCGA-CV-7250-01	P38S	Missense_Mutation	Somatic
Head and Neck Squamous Cell Carcinoma (TCGA, PanCancer Atlas	TCGA-CV-7250-01	P38S	Missense_Mutation	.
TCGA data for Esophagus-Stomach Cancers (TCGA)	TCGA-BR-4361-01	P38Rfs*34	Frame_Shift_Del	Somatic
Stomach Adenocarcinoma (TCGA)	TCGA-BR-4361-01	P38Rfs*34	Frame_Shift_Del	Somatic
Stomach Adenocarcinoma (TCGA, Provisional)	TCGA-BR-4361-01	P38Rfs*34	Frame_Shift_Del	Somatic
Stomach Adenocarcinoma (TCGA, PanCancer Atlas)	TCGA-BR-4361-01	P38Rfs*34	Frame_Shift_Del	.
TCGA data for Esophagus-Stomach Cancers (TCGA)	TCGA-BR-8363-01	E14K	Missense_Mutation	Somatic
Stomach Adenocarcinoma (TCGA,)	TCGA-BR-8363-01	E14K	Missense_Mutation	Unknown
Stomach Adenocarcinoma (TCGA, Provisional)	TCGA-BR-8363-01	E14K	Missense_Mutation	Somatic
Stomach Adenocarcinoma (TCGA, PanCancer Atlas)	TCGA-BR-8363-01	E14K	Missense_Mutation	.
Stomach Adenocarcinoma (UHK)	pfg016T	S3N	Missense_Mutation	NA
TCGA data for Esophagus-Stomach Cancers (TCGA)	TCGA-BR-A4PE-01	S3N	Missense_Mutation	Somatic
Stomach Adenocarcinoma (TCGA)	TCGA-BR-A4PE-01	S3N	Missense_Mutation	Unknown
Stomach Adenocarcinoma (TCGA, Provisional)	TCGA-BR-A4PE-01	S3N	Missense_Mutation	Somatic
Stomach Adenocarcinoma (TCGA, PanCancer Atlas)	TCGA-BR-A4PE-01	S3N	Missense_Mutation	.
Head and Neck Squamous Cell Carcinoma (TCGA)	TCGA-CV-5441-01	P28L	Missense_Mutation	Unknown
Head and Neck Squamous Cell Carcinoma (TCGA, Provisional)	TCGA-CV-5441-01	P28L	Missense_Mutation	Somatic
Head and Neck Squamous Cell Carcinoma (TCGA, PanCancer Atlas)	TCGA-CV-5441-01	P28L	Missense_Mutation	.
Skin Cutaneous Melanoma (TCGA, Provisional)	TCGA-ER-A3EV-06	E15del	In_Frame_Del	Somatic
Skin Cutaneous Melanoma (Broad)	Pat_08_Pre	E15del	In_Frame_Del	NA
Skin Cutaneous Melanoma (Broad)	Pat_40_Post	E15del	In_Frame_Del	NA
Skin Cutaneous Melanoma (Broad)	Pat_70_Post	E15del	In_Frame_Del	NA
Bladder Urothelial Carcinoma (TCGA)	TCGA-BT-A3PH-01	P28S	Missense_Mutation	NA
Bladder Cancer (TCGA)	TCGA-BT-A3PH-01	P28S	Missense_Mutation	Somatic
Bladder Urothelial Carcinoma (TCGA)	TCGA-BT-A3PH-01	P28S	Missense_Mutation	Somatic
Bladder Urothelial Carcinoma (TCGA)	TCGA-BT-A3PH-01	P28S	Missense_Mutation	.
Hepatocellular Carcinomas (Inserm)	CHC892T	A149T	Missense_Mutation	NA
Whole-exome sequences (WES) of pretreatment melanoma tumors (UCLA)	Pt1	L173Qfs*3	Frame_Shift_Ins	NA
Colorectal Adenocarcinoma (Genentech)	587338	G193D	Missense_Mutation	NA
Colorectal Adenocarcinoma (Genentech)	587368	P196A	Missense_Mutation	NA
NCI-60 Cell Lines (NCI)	MDA_MB_435	G139R	Missense_Mutation	NA
NCI-60 Cell Lines (NCI)	MDA_N	G139R	Missense_Mutation	NA
Multiple Myeloma (Broad)	MM-0478	G197D	Missense_Mutation	NA
Pancreatic Adenocarcinoma (QCMG)	ICGC_0013	E15-	Targeted_Region	Somatic
Pancreatic Adenocarcinoma (QCMG)	ICGC_0076	EK15EK	Frame_Shift_Ins	Somatic
Pancreatic Adenocarcinoma (TCGA)	TCGA-IB-7651-01	V90M	Missense_Mutation	.
Pancreatic Adenocarcinoma (TCGA, Provisional)	TCGA-IB-7651-01	V90M	Missense_Mutation	Somatic
Prostate Adenocarcinoma (MSKCC/DFCI)	SC_9171	A85V	Missense_Mutation	NA

Skin Cutaneous Melanoma (TCGA, Provisional)	TCGA-ER-A19D-06	A85V	Missense_Mutation	Somatic
Skin Cutaneous Melanoma (TCGA, PanCancer Atlas)	TCGA-ER-A19D-06	A85V	Missense_Mutation	.
Bladder Cancer (TCGA)	TCGA-G2-AA3B-01	S49*	Nonsense_Mutation	Somatic
Bladder Urothelial Carcinoma (TCGA, PanCancer Atlas)	TCGA-G2-AA3B-01	S49*	Nonsense_Mutation	.
Cervical Squamous Cell Carcinoma (TCGA, PanCancer Atlas)	TCGA-MA-AA42-01	R76C	Missense_Mutation	.
Glioblastoma Multiforme (TCGA, PanCancer Atlas)	TCGA-06-5416-01	R76C	Missense_Mutation	.
Adrenocortical Carcinoma (TCGA, Provisional)	TCGA-OR-A5KB-01	S114F	Missense_Mutation	Somatic
Adrenocortical Carcinoma (TCGA, PanCancer Atlas)	TCGA-OR-A5KB-01	S114F	Missense_Mutation	.
Head and Neck Squamous Cell Carcinoma (TCGA, Provisional)	TCGA-CV-7568-01	A39T	Missense_Mutation	Somatic
Head and Neck Squamous Cell Carcinoma (TCGA, PanCancer Atlas)	TCGA-CV-7568-01	A39T	Missense_Mutation	.
Low-Grade Gliomas (UCSF)	P21_Rec	V99M	Missense_Mutation	NA
Low-Grade Gliomas (UCSF)	P24_Rec2	P134L	Missense_Mutation	NA
Prostate Adenocarcinoma (MSKCC/DFCI)	SC_9130	L173M	Missense_Mutation	NA
Breast Invasive Carcinoma (TCGA, PanCancer Atlas)	TCGA-A8-A07C-01	V223_P234del	In_Frame_Del	.
Breast Invasive Carcinoma (TCGA, PanCancer Atlas)	TCGA-AN-A04C-01	COX6B1-SERTAD1	Fusion	NA
Lung Squamous Cell Carcinoma (TCGA, PanCancer Atlas)	TCGA-21-1080-01	L64M	Missense_Mutation	.
Ovarian Serous Cystadenocarcinoma (TCGA, PanCancer Atlas)	TCGA-24-2280-01	CLSTN1-SERTAD1	Fusion	NA
Skin Cutaneous Melanoma (TCGA, PanCancer Atlas)	TCGA-D3-A2JP-06	P38T	Missense_Mutation	.
Skin Cutaneous Melanoma (TCGA, PanCancer Atlas)	TCGA-D3-A3CE-06	P38Q	Missense_Mutation	.
Skin Cutaneous Melanoma (TCGA, PanCancer Atlas)	TCGA-EE-A29S-06	R8L	Missense_Mutation	.
Skin Cutaneous Melanoma (TCGA, PanCancer Atlas)	TCGA-W3-A824-06	L48P	Missense_Mutation	.
The Metastatic Breast Cancer Project (Provisional)	MBC-MBCProject_9JI1fwuX-Tumor-SM-DL4TP	S49*	Nonsense_Mutation	Somatic
Colorectal Adenocarcinoma (DFC,)	coadread_dfci_2016_189255	A149V	Missense_Mutation	NA
Colorectal Adenocarcinoma (DFCI)	coadread_dfci_2016_2417	D22N	Missense_Mutation	NA
Pediatric Pan-Cancer (DKFZ-German Cancer Consortium)	ALL-B-5-R	A84T	Missense_Mutation	NA
Mixed Tumors (PIP-Seq 2017)	PIP13-95124-T1	T31A	Missense_Mutation	NA
Mixed Tumors (PIP-Seq 2017)	PIP13-95124-T2	T31A	Missense_Mutation	NA
Mixed Tumors (PIP-Seq 2017)	PIP15-70532-T2	T31A	Missense_Mutation	NA
Cutaneous Squamous Cell Carcinoma (MD Anderson)	CSCC-27-T	P89L	Missense_Mutation	Somatic
Adrenocortical Carcinoma (TCGA, PanCancer Atlas)	TCGA-OR-A5K5-01	KCNMA1-SERTAD1	Fusion	NA
Colon Adenocarcinoma (TCGA, PanCancer Atlas)	TCGA-A6-3809-01	N184H	Missense_Mutation	.
Colon Adenocarcinoma (TCGA, PanCancer Atlas)	TCGA-AD-6889-01	A20V	Missense_Mutation	.
Colon Adenocarcinoma (TCGA, PanCancer Atlas)	TCGA-AZ-4315-01	E214K	Missense_Mutation	.
Lung Adenocarcinoma (TCGA, PanCancer Atlas)	TCGA-95-7043-01	G233W	Missense_Mutation	.

[1] All studies for mutational analysis of SERTAD1 in various types of cancers datas have been retrieved from http://www.cbioportal.org/ by following the previous literature [30].

Table S3. Tissue and cancer specific biological networks depicted significant correlation network between breast cancer and normal tissue.

	Breast Cancer (BRAC)			Normal Breast tissue		
**S. No.**	**ID Source Node**	**ID Target Node**	**Score**	**ID Source Node**	**ID Target Node**	**Score**
1	SERTAD1	JOSD2	0.615	SERTAD1	UBE2S	0.588
2	SERTAD1	RBM42	0.592	SERTAD1	PNO1	0.552
3	SERTAD1	LAMTOR4	0.576	SERTAD1	PSMC4	0.542
4	SERTAD1	SDHAF1	0.575	SERTAD1	BCL3	0.532
5	SERTAD1	RABAC1	0.573	SERTAD1	NXT1	0.530
6	SERTAD1	MYL6	0.570	SERTAD1	TSSC4	0.529
7	SERTAD1	NOSIP	0.565	SERTAD1	TNIP2	0.522
8	SERTAD1	INAFM1	0.564	SERTAD1	CLTB	0.521
9	SERTAD1	FIS1	0.562	SERTAD1	SNRPD1	0.518
10	SERTAD1	ZNF524	0.552	SERTAD1	TEAD4	0.517
11	SERTAD1	SELENOW	0.552	SERTAD1	ATG101	0.514
12	SERTAD1	DAPK3	0.552	SERTAD1	BAG3	0.514
13	SERTAD1	EIF3K	0.551	SERTAD1	SH2B2	0.512
14	SERTAD1	MRPL54	0.548	SERTAD1	BATF3	0.509
15	SERTAD1	ALKBH7	0.547	SERTAD1	PNP	0.507
16	SERTAD1	CCDC61	0.546	SERTAD1	CCDC85B	0.503
17	SERTAD1	TSR3	0.546	SERTAD1	MAP2K3	0.502
18	SERTAD1	BCL7C	0.545	SERTAD1	MFHAS1	0.501
19	SERTAD1	LMNA	0.541	SERTAD1	PPRC1	0.498
20	SERTAD1	PHLDA3	0.540	SERTAD1	NTMT1	0.496

Table S4. Tissue and cancer specific biological networks depicted significant correlation network between Melanoma and normal skin tissue.

	Melanoma (CMM)			Normal Skin Tissue		
**S. No.**	**ID Source Node**	**ID Target Node**	**Score**	**ID Source Node**	**ID Target Node**	**Score**
1	SERTAD1	DEDD2	0.687	SERTAD1	PHLDA2	0.578
2	SERTAD1	PSMB6	0.676	SERTAD1	SLC25A25	0.568
3	SERTAD1	SH3BGRL3	0.666	SERTAD1	HBEGF	0.541
4	SERTAD1	C19orf47	0.663	SERTAD1	FOSL1	0.525
5	SERTAD1	MSRB1	0.650	SERTAD1	TGIF1	0.520
6	SERTAD1	SERTAD3	0.634	SERTAD1	IER5	0.518
7	SERTAD1	MYL6	0.630	SERTAD1	ID1	0.517
8	SERTAD1	ICE2	−0.629	SERTAD1	SLC20A1	0.515
9	SERTAD1	TADA1	−0.624	SERTAD1	DNAJB1	0.500
10	SERTAD1	ITPKC	0.614	SERTAD1	ZNF593	0.500
11	SERTAD1	BCL3	0.595	SERTAD1	HSPA1B	0.499
12	SERTAD1	AP2S1	0.593			
13	SERTAD1	GIPC1	0.592			
14	SERTAD1	SHKBP1	0.592			
15	SERTAD1	TECR	0.589			
16	SERTAD1	KLHL24	−0.587			
17	SERTAD1	MT2A	0.587			
18	SERTAD1	BORCS6	0.581			

Table S5. Tissue and cancer specific biological networks depicted significant correlation network between Liver cancer and normal liver tissue.

	Liver Cancer (HCC)			Normal Liver Tissue		
**S. No.**	**ID Source Node**	**ID Target Node**	**Score**	**ID Source Node**	**ID Target Node**	**Score**
1	SERTAD1	IER2	0.521	SERTAD1	PLEKHB2	0.832
2	SERTAD1	SERTAD3	0.519	SERTAD1	GPAT3	0.827
3	SERTAD1	JUNB	0.508	SERTAD1	MAFF	0.816
4	SERTAD1	PLK3	0.503	SERTAD1	ITPKC	0.809
5	SERTAD1	C11orf96	0.478	SERTAD1	MCL1	0.806
6	SERTAD1	ID1	0.474	SERTAD1	EPHA2	0.805
7	SERTAD1	PLEKHA4	0.445	SERTAD1	ELMSAN1	0.805
8	SERTAD1	ITPKC	0.438	SERTAD1	JOSD1	0.803
9	SERTAD1	ATL2	-0.429	SERTAD1	CREM	0.799
10	SERTAD1	RABAC1	0.428	SERTAD1	ZSWIM6	0.796
11	SERTAD1	MYL6	0.425	SERTAD1	DUSP5	0.792

Table S6. SERTAD1 expression and alteration versus cancer patient's prognosis.

Study	Patients (N)/Samples	Data Type	Expression/Alteration	Survival (OS or DFS)	Prognosis
Mixed Tumor (PIP-Seq 2017 [30])	88/103	Expression & Mutation	Altered (2.3%)	OS	Worse
Breast Invasive Carcinoma (TCGA, *cell* 2015 [61])	816/818	Expression & CNA	Altered (2%)	OS and DFS	Worse
Breast Invasive Carcinoma (TCGA, provisional [30])	1101/1108	Expression & CNA	Altered (2.4%)	OS and DFS	Worse
Merged Cohort of LGG & GBM (TCGA, *cell* 2016 [62])	794/1122	Expression & CNA	Altered (1%)	OS	Better
Pan-Lung Cancer (TCGA, *Nat. Genet.* 2016 [63])	954/1144	Expression & CNA	Altered (4%)	OS	Worse
Cutaneous Squamous Cell Carcinoma (MD Anderson [30])	38/39	Expression & Mutation	Altered (3%)	OS	Worse
TCGA data for Esophagus-Stomach Cancer (TCGA [30])	265/559	Expression & CNA	Altered (4%)	OS	Better

Table S7. Associated protein network involved in various types of pathways.

Pathway ID	Pathway Description	CGS	FDR
04110	Cell cycle	6	0.000000239
05166	HTLV-I-infection	7	0.000000239
05219	Bladder cancer	3	0.000495
05200	Pathways in cancer	5	0.000506
05206	MicroRNAs in cancer	4	0.000506
05203	Viral carcinogenesis	4	0.000753
05212	Pancreatic cancer	3	0.000753
05214	Glioma	3	0.000753
05223	Non-small cell lung cancer	3	
04115	P53 signaling pathway	3	0.000819
05218	Melanoma	3	0.000819
05220	Chronic myeloid leukemia	3	0.000819
04350	TGF-beta signaling pathway	3	0.00105
05222	Small cell lung cancer	3	0.00127
05161	Hepatitis B	3	0.00526
05215	Prostate cancer	2	0.05

FDR = False discovery rate, CGS = Count in gene set.

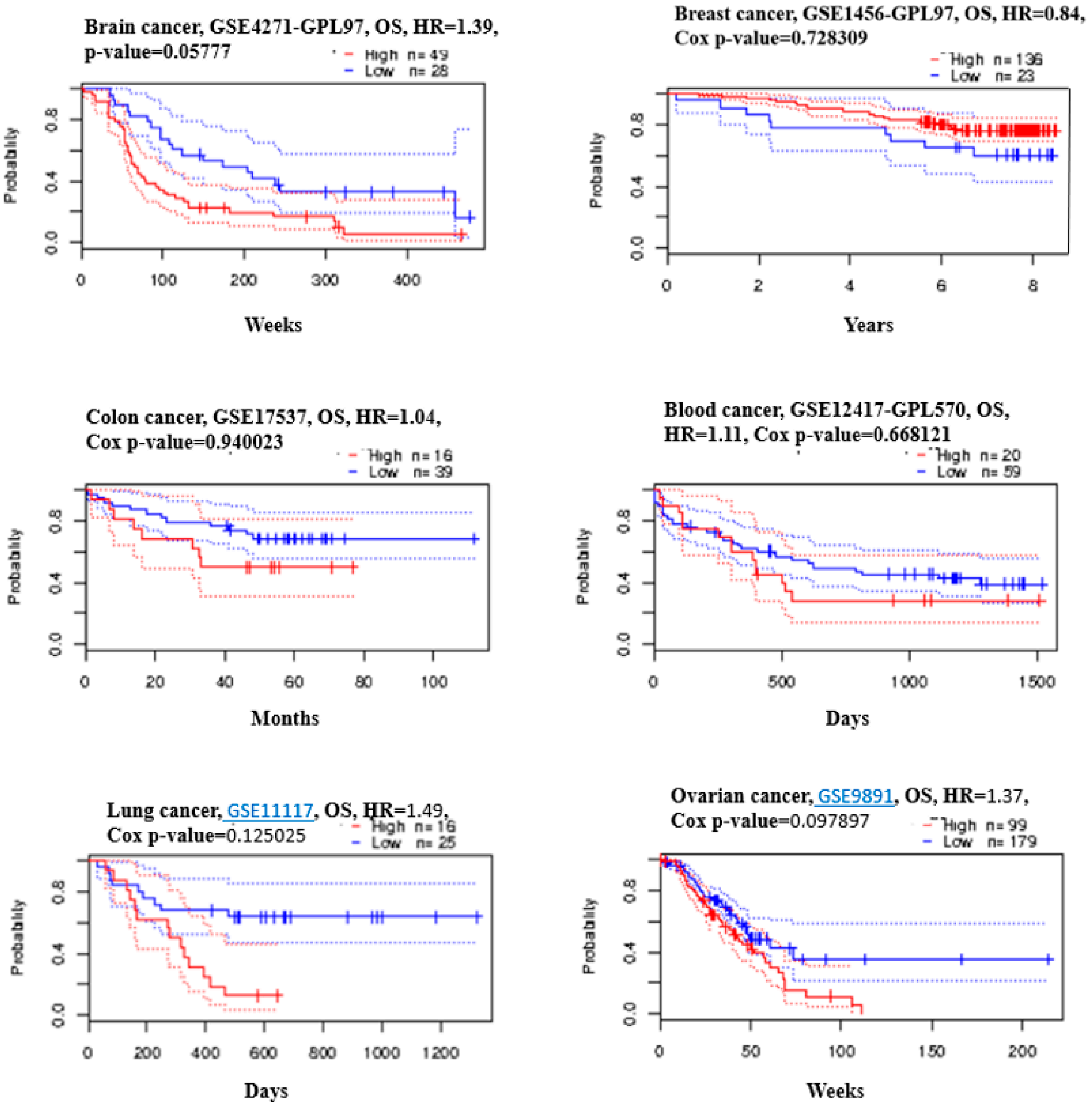

Figure S1. Relationship between SERTAD1 expression and patient outcome in different cancer patient's survival analysis.

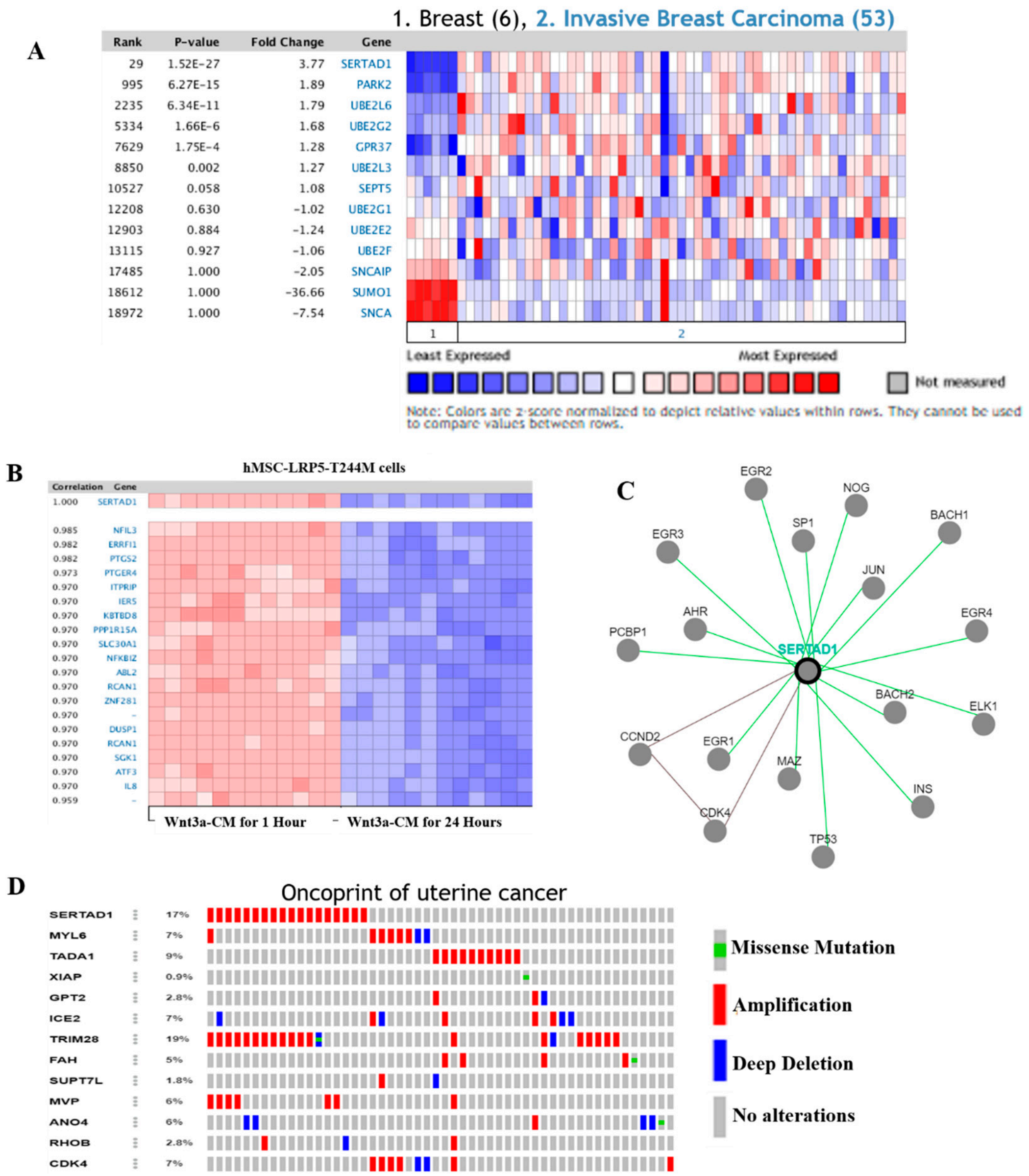

Figure S2. Putative relationship and co-expression of SERTAD1 with various candidate factors. A. Differential expression of SERTAD1 with other oncogenes. **A**. Normal vs tumor cells of invasive breast cancer vs normal mammary cells. Identified subsets of genes differentially expressed in human invasive breast cancer versus normal breast tissue. More than 1000 genes significantly linked with invasiveness of breast cancer. Especially SERTAD1 and Parkin2 shown similar expression pattern in cancers tissue as compared to normal. Analysis clearly showed that expression levels in fold change with significant p-value and gene rank among all genes. **B**. Common pathway analysis predicted co-association of SERTAD1 with TP53, XIAP and so on. **C**. Oncoprint analysis showed SERTAD1 simultaneously mutated with MYL6, TADA1, XIAP, and other genes in uterine cancer.

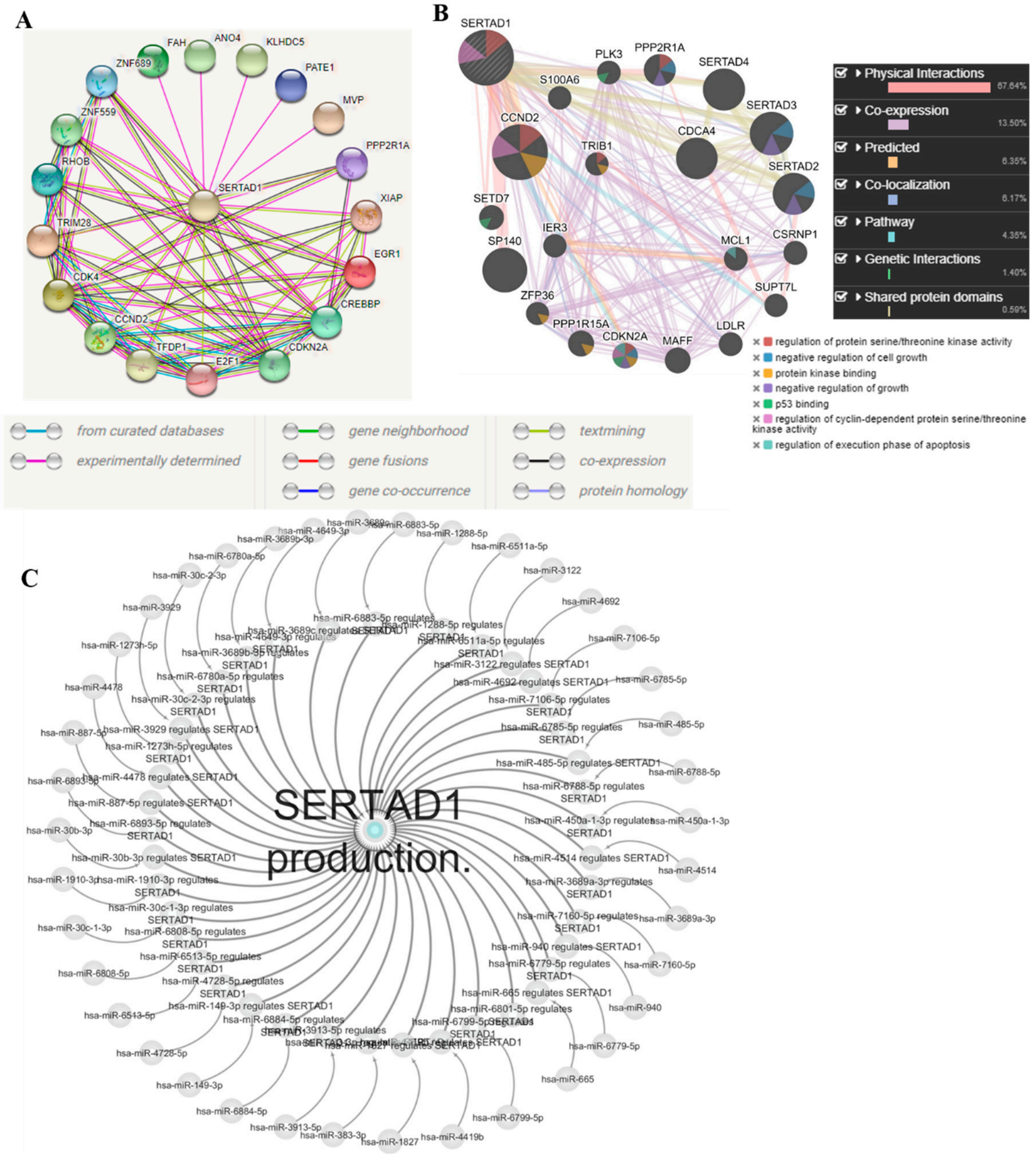

Figure S3. The bridge avenue model for SERTAD1 association and its relationship with genes, proteins and miRNAs. **A**. The 20 genes most related to human SERTAD1, based on GEO dataset. The expression profiles from this dataset were converted into a co-expression network using the GeneMANIA app. **B**. An integrated protein-protein interaction network is combined with tumor initiating proteomic profiles to dissect relationship densely connected proteins in different

cell program. We estimated the false discovery rate (FDR) with 10% that depicted various pathways. **C**. Schematic depiction of several microRNAs (miRNAs) are participating in transcription processes of SERTAD1 and regulate cellular signaling pathways. The network is generated by common pathways analysis online tool.

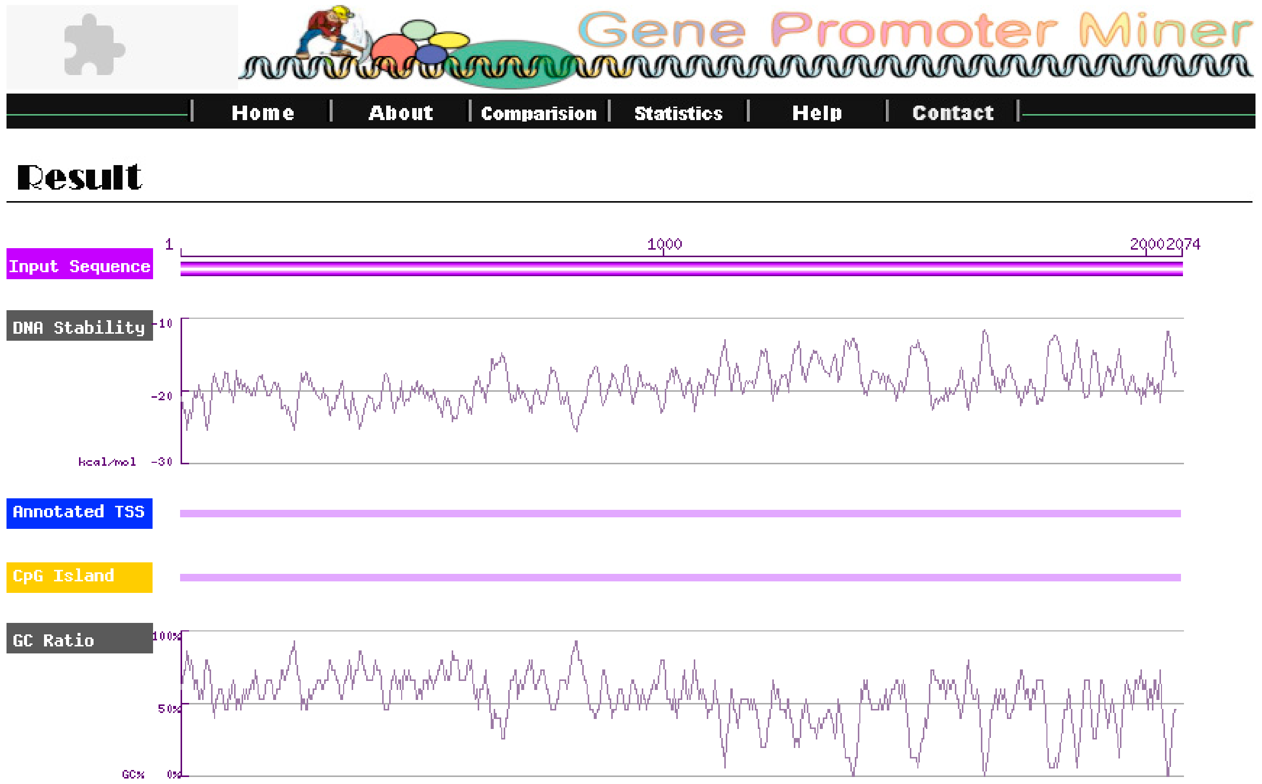

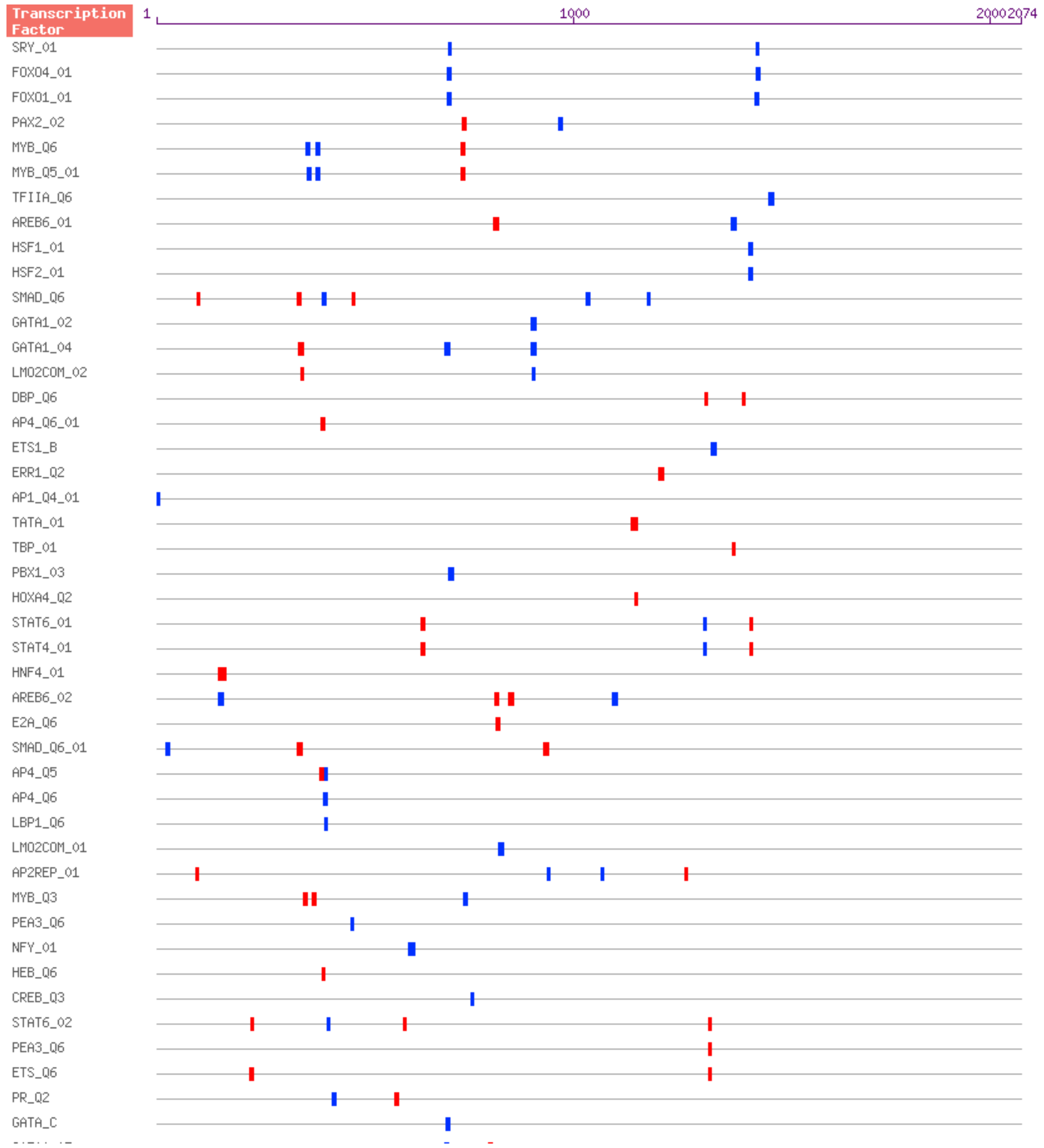
Transcription Factor
1
1000
2000
2074
SRY_01
FOXO4_01
FOXO1_01
PAX2_02
MYB_Q6
MYB_Q5_01
TFIIA_Q6
AREB6_01
HSF1_01
HSF2_01
SMAD_Q6
GATA1_02
GATA1_04
LMO2COM_02
DBP_Q6
AP4_Q6_01
ETS1_B
ERR1_Q2
AP1_Q4_01
TATA_01
TBP_01
PBX1_03
HOXA4_Q2
STAT6_01
STAT4_01
HNF4_01
AREB6_02
E2A_Q6
SMAD_Q6_01
AP4_Q5
AP4_Q6
LBP1_Q6
LMO2COM_01
AP2REP_01
MYB_Q3
PEA3_Q6
NFY_01
HEB_Q6
CREB_Q3
STAT6_02
PEA3_Q6
ETS_Q6
PR_Q2
GATA_C

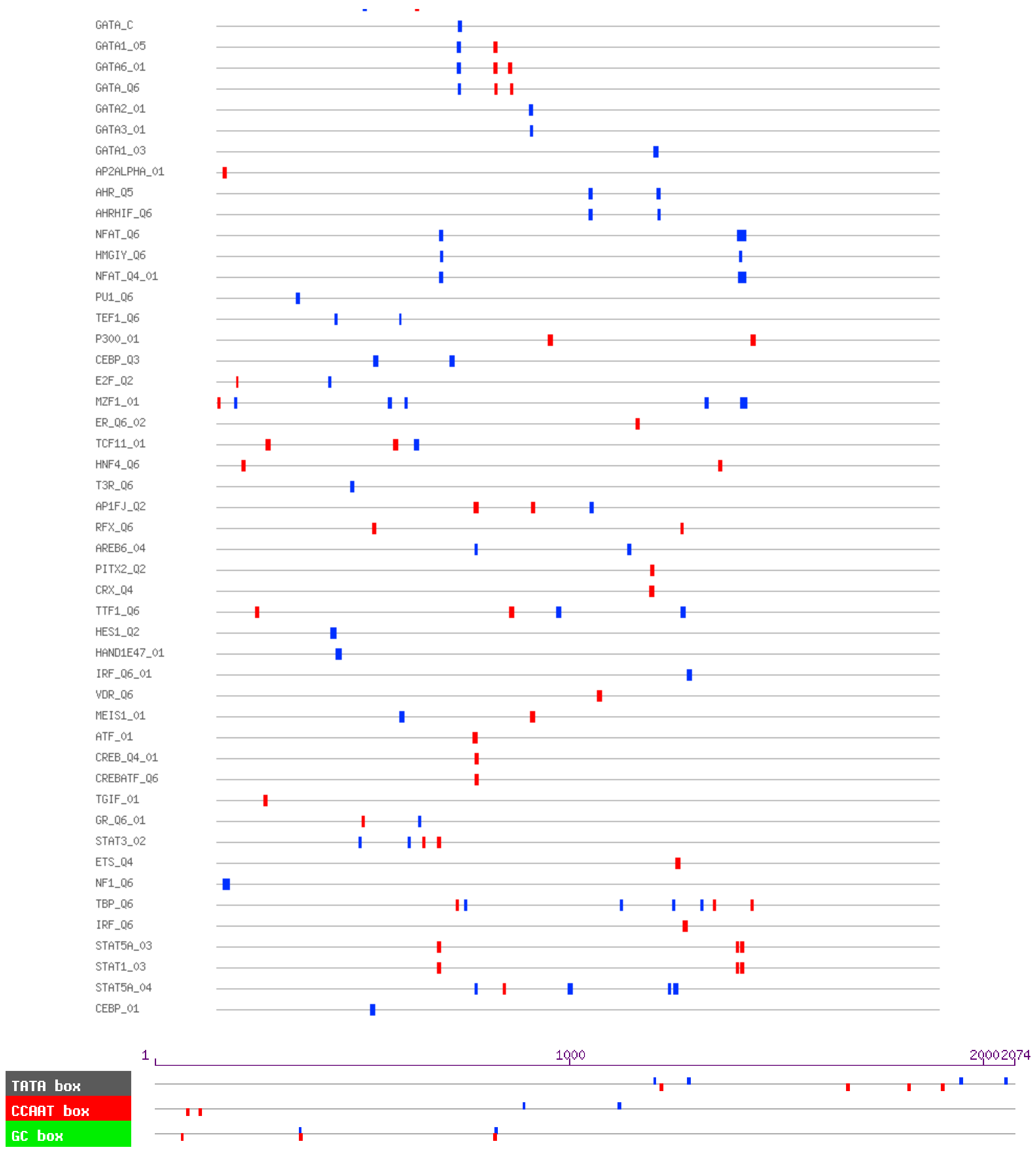
GATA_C
GATA1_05
GATA6_01
GATA_Q6
GATA2_01
GATA3_01
GATA1_03
AP2ALPHA_01
AHR_Q5
AHRHIF_Q6
NFAT_Q6
HMGIY_Q6
NFAT_Q4_01
PU1_Q6
TEF1_Q6
P300_01
CEBP_Q3
E2F_Q2
MZF1_01
ER_Q6_02
TCF11_01
HNF4_Q6
T3R_Q6
AP1FJ_Q2
RFX_Q6
AREB6_04
PITX2_Q2
CRX_Q4
TTF1_Q6
HES1_Q2
HAND1E47_01
IRF_Q6_01
VDR_Q6
MEIS1_01
ATF_01
CREB_Q4_01
CREBATF_Q6
TGIF_01
GR_Q6_01
STAT3_02
ETS_Q4
NF1_Q6
TBP_Q6
IRF_Q6
STAT5A_03
STAT1_03
STAT5A_04
CEBP_01
1
1000
2000
2074
TATA box
CCAAT box
GC box

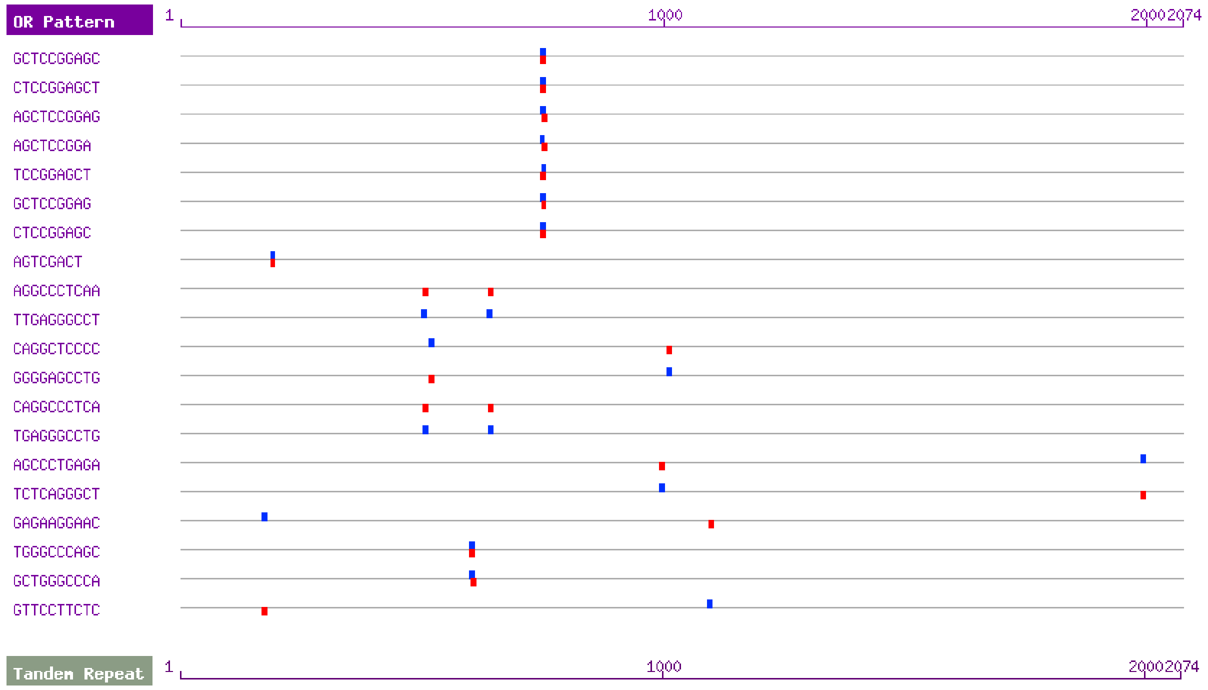

Figure S4. Transcription factor profiling of SERTAD1 promoter region.

© 2019 by the authors. Licensee MDPI, Basel, Switzerland. This article is an open access article distributed under the terms and conditions of the Creative Commons Attribution (CC BY) license (http://creativecommons.org/licenses/by/4.0/).
